# Trends in carbapenem antibiotics utilization, costs, and market dynamics in Medicaid: a retrospective analysis from 1991 to 2023

**DOI:** 10.3389/fmed.2025.1589981

**Published:** 2025-08-07

**Authors:** Marwan A. Alrasheed, Abdulrahman A. Alsuhibani

**Affiliations:** ^1^Department of Clinical Pharmacy, College of Pharmacy, King Saud University, Riyadh, Saudi Arabia; ^2^Department of Pharmacy Practice, College of Pharmacy, Qassim University, Qassim, Saudi Arabia

**Keywords:** carbapenem, antibiotics utilization, spending, trends, COVID-19, Medicaid

## Abstract

**Background:**

Carbapenems are vital antibiotics used to treat severe infections, particularly those caused by multidrug-resistant organisms. Understanding their utilization, reimbursement, and pricing trends is critical to inform healthcare policies and ensure sustainable access. This study aimed to analyze longitudinal trends in carbapenem use, market dynamics, and economic impact within Medicaid from 1991 to 2023.

**Methods:**

A retrospective, longitudinal analysis was conducted using publicly available Medicaid State Drug Utilization data. Four carbapenems; imipenem/cilastatin, meropenem, ertapenem, and doripenem were examined. Data on prescriptions, reimbursements, and proxy pricing were extracted, aggregated, and analyzed. Trends and market shares were evaluated annually, with statistical analyses and visualizations performed using Excel and Power BI. Joinpoint regression analysis was used to identify shifts in the direction of these trends, providing insights into significant changes over time.

**Results:**

Carbapenem utilization and reimbursement exhibited significant growth, particularly between 2015 and 2020, driven by increased reliance on these agents for severe infections. Imipenem/cilastatin prescriptions peaked in 2005 at 22,883 prescriptions, with an Annual Percent Change (APC) of 12.28% (95% CI: 11.33 to 14.03%, *p* < 0.001). Reimbursements for imipenem/cilastatin reached $7.6 million in the same year. Following 2005, a sharp decline in prescriptions occurred, with an APC of −30.36% (95% CI: −33.96% to −25.44%, *p* < 0.001), and reimbursements dropped to $839,581 by 2023. Meropenem prescriptions exhibited steady growth, peaking at 53,355 in 2016, with an Average Annual Percent Change (AAPC) of 17.29% (95% CI: 13.97 to 29.71%, *p* < 0.001). Reimbursements for meropenem reached $10.9 million in 2016 before stabilizing at $7.2 million in 2023. Ertapenem, introduced in 2002, demonstrated the most substantial growth, peaking in 2021 with 116,988 prescriptions and $30.7 million in reimbursements. Its APC between 2002 and 2015 was 19.05% (95% CI: 13.56 to 109.30%, *p* = 0.023). Doripenem prescriptions peaked at 2,954 in 2011 but declined sharply at an APC of −39.24% (95% CI: −52.08% to −35.15%, *p* < 0.001), with reimbursements dropping to negligible levels by 2017. Declines in carbapenem prescriptions and reimbursements after 2021 reflect antimicrobial stewardship efforts and shifts toward targeted therapies.

**Conclusion:**

This study underscores dynamic shifts in carbapenem utilization, highlighting the impact of clinical practices, stewardship initiatives, and policy changes on prescribing trends. Continuous monitoring of antibiotic use is essential to balance effective infection management with the containment of antimicrobial resistance and healthcare costs. Further research is needed to evaluate the long-term effects of stewardship programs and the introduction of novel therapeutic options on carbapenem utilization.

## Introduction

Carbapenems are a vital subclass of beta-lactam antibiotics, characterized by their unique beta-lactam ring structure. The broad-spectrum activity of this class of antibiotics, which also includes penicillins, monobactams, and cephalosporins, stands out for its broad-spectrum activity. Numerous multidrug-resistant species that provide serious challenges in clinical settings are among the many bacterial infections that carbapenems are especially efficient against (1).

Ertapenem, imipenem, meropenem, and doripenem are notable carbapenem agents. Since their chemical makeup makes them unsuitable for oral absorption, these antibiotics are given parenterally. Because of their strong antibacterial activity, carbapenems are frequently used in combination with aminoglycosides to treat severe or complicated infections. This synergistic approach enhances the efficacy of both drugs, providing a robust defense against challenging bacterial infections (2).

Imipenem’s special formulation includes cilastatin, a substance that prevents renal dehydropeptidase from breaking it down, extending its therapeutic action (3). In some cases, imipenem is further combined with relebactam, a beta-lactamase inhibitor, to combat resistant bacterial strains. These combinations underscore the innovative approaches in optimizing carbapenem efficacy and extending their clinical utility (4, 5).

In today’s healthcare system, carbapenems are crucial in treating severe infections, especially those caused by multidrug-resistant organisms. However, their increased use has led to rising resistance, emphasizing the need for robust antimicrobial stewardship programs. The Infectious Diseases Society of America (IDSA) provides guidance on managing infections caused by resistant Gram-negative bacteria, highlighting the importance of appropriate carbapenem use (6). Furthermore, understanding trends in carbapenem utilization, costs, and pricing is crucial for informing healthcare policies and ensuring sustainable access to these life-saving medications (7–10). This study aims to explore these aspects within Medicaid programs from 1991 to 2023, shedding light on the clinical and economic impact of carbapenem antibiotics.

## Methods

### Study design and data source

This was a retrospective, longitudinal analysis of prescription, reimbursement, and pricing trends for carbapenem antibiotics in the Medicaid program from 1991 to 2023. The study utilized publicly available Medicaid State Drug Utilization data, which includes detailed information on the number of prescriptions, reimbursement amounts, and unit prices for drugs dispensed under the Medicaid program.

### Study population

The study focused on four carbapenem antibiotics: Imipenem/cilastatin, meropenem, ertapenem, and doripenem. Data were aggregated across all states in the United States where Medicaid utilization data were available. The study population included all Medicaid beneficiaries who received prescriptions for these carbapenems during the study period.

### Data collection

Data were extracted for the number of prescriptions, total reimbursement, and proxy pricing (calculated as total reimbursement divided by the number of prescriptions) for each carbapenem agent. Market share percentages for prescriptions and reimbursement were calculated as the proportion of each agent relative to the total for all four carbapenems in a given year. The analysis was stratified by year to assess longitudinal trends.

### Data analysis

The primary outcomes were:

*Prescriptions*: Trends in the annual number of prescriptions and market share percentages for each carbapenem.
*Reimbursement*: Annual total reimbursement amounts and market share percentages.
*Proxy Price*: The annual proxy price for each agent, serving as an estimate of drug cost per prescription.

Descriptive statistics were used to summarize the data. Trends were visualized using line graphs to illustrate changes over time. Market share percentages for prescriptions and reimbursement were calculated by dividing the values for each carbapenem by the total across all agents for each year. Data cleaning and processing were conducted using Excel, and visualizations were generated using Power BI.

### Ethical considerations

The study utilized publicly available, de-identified data from the Medicaid program, ensuring there were no risks to patient confidentiality or privacy. As no human subjects were involved, institutional review board (IRB) approval was not required.

## Results

Cumulative carbapenem prescriptions increased steadily from 1991 to 2010, followed by a sharp rise between 2015 and 2020. The highest number of prescriptions was recorded in 2021, after which a slight decline was observed. Similarly, cumulative reimbursements showed consistent growth over the years, with a significant surge from 2015 to 2020, peaking in 2021. This peak was followed by a noticeable decline in reimbursement amounts in the subsequent years. Both prescriptions and reimbursements demonstrated parallel growth patterns, with the most substantial increases occurring in the last decade (Supplementary Table 1).

### Imipenem-Cilastatin

#### Imipenem-Cilastatin utilization in Medicaid (1991–2023)

**Figure 1:**
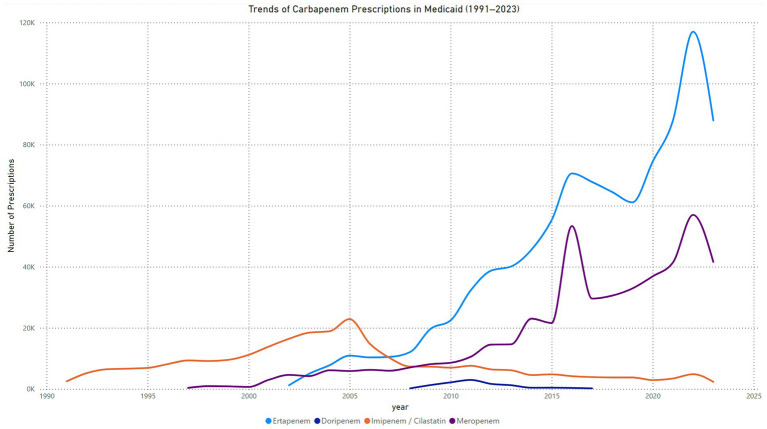
Trend in carbapenems prescriptions in Medicaid.

The quarterly trends in Imipenem-Cilastatin prescriptions in Medicaid from 1991 to 2023 reveal significant fluctuations over time. Prescription volumes increased steadily from 1991, peaking between 2001 and 2005, with the highest recorded quarterly volume exceeding 6,000 prescriptions. After 2005, a sharp decline in prescription volumes was observed, continuing through 2015. Between 2016 and 2023, prescription volumes stabilized at lower levels, averaging fewer than 1,000 prescriptions per quarter. Periodic spikes were noted in some quarters, such as in 2021 (Figure 1).

The Joinpoint Regression analysis identified significant trends in imipenem/cilastatin prescriptions from 1991 to 2023. Between 1991 and 2005, the Annual Percent Change (APC) was 12.28% (95% CI: 11.33 to 14.03%, *p* < 0.000001). From 2005 to 2008, a sharp decline was observed, with an APC of −30.36% (95% CI: −33.96% to −25.44%, *p* < 0.000001). From 2008 to 2023, the APC was −5.38% (95% CI: −7.73% to −2.96%, *p* = 0.0036). The Average Annual Percent Change (AAPC) for the entire period was −0.91% (95% CI: −1.90% to −0.14%, *p* = 0.0260) (Supplementary material).

#### Imipenem-Cilastatin reimbursement in Medicaid (1991–2023)

**Figure 2:**
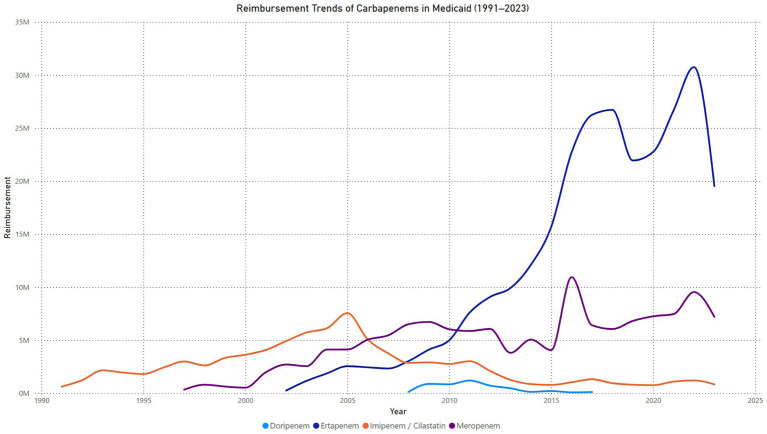
Trend in carbapenems reimbursement in Medicaid.

The quarterly reimbursement trends for Imipenem-Cilastatin in Medicaid from 1991 to 2023 demonstrate substantial variability over the analysis period. Reimbursement amounts steadily increased from 1991, peaking between 2004 and 2005, with quarterly reimbursements exceeding $2 million. Following 2005, a sharp decline in reimbursement amounts was observed, with values steadily decreasing through 2015. Between 2016 and 2023, reimbursement levels stabilized at significantly lower amounts, averaging less than $500,000 per quarter. Isolated spikes in reimbursement were evident in certain quarters, such as in 2021 (Figure 2).

The Joinpoint Regression analysis identified significant trends in imipenem/cilastatin reimbursement from 1991 to 2023. From 1991 to 2005, the APC was 13.23% (95% CI: 12.32 to 14.75%, *p* = 0.0012). Between 2005 and 2008, a significant decline was observed, with an APC of −27.32% (95% CI: −30.85% to −24.24%, *p* = 0.0104). From 2008 to 2011, there was no statistically significant change, with an APC of 2.53% (95% CI: −4.98 to 9.39%, *p* = 0.4847). A sharp decline occurred between 2011 and 2014, with an APC of −31.47% (95% CI: −39.41% to −19.45%, *p* = 0.0016). From 2014 to 2023, the trend showed no significant change, with an APC of 1.18% (95% CI: −3.93 to 13.51%, *p* = 0.4843). The AAPC for the entire period was −0.53% (95% CI: −1.67 to 0.67%, *p* = 0.3347), indicating no statistically significant overall change in reimbursement trends (Supplementary material).

#### Imipenem-Cilastatin price in Medicaid (1991–2023)

**Figure 3:**
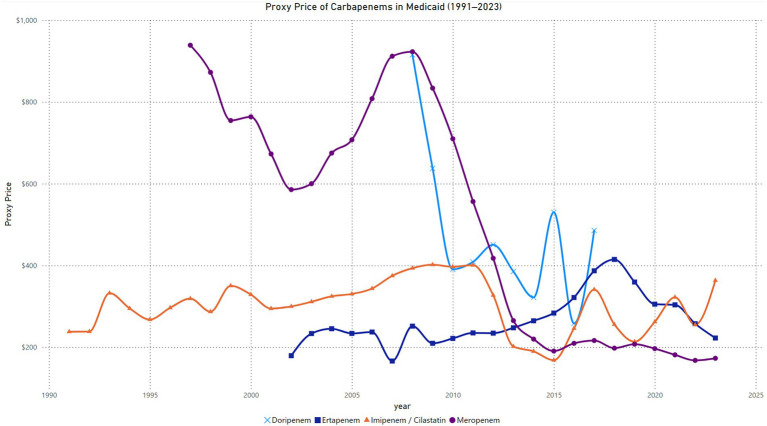
Trend in carbapenem price in Medicaid.

The quarterly proxy price trends for Imipenem-Cilastatin in Medicaid from 1991 to 2023 reveal distinct phases of variability. Prices began near $200 per unit in 1991, with a significant spike reaching approximately $650 per unit in 1993, the highest recorded value. Between 1994 and 2011, prices stabilized within the range of $300 to $500 per unit, with periodic fluctuations. A gradual decline in prices began in 2012, with values falling below $300 per unit by 2015, marking the lowest sustained levels during the analysis period. From 2016 onward, proxy prices exhibited a steady upward trend, reaching approximately $500 per unit by 2023. This upward trajectory was accompanied by periodic oscillations, reflecting variability within the overall rising pattern (Figure 3).

The Joinpoint Regression analysis identified significant trends in imipenem/cilastatin prices from 1991 to 2023. From 1991 to 2011, the APC was 2.01% (95% CI: 1.22 to 3.26%, *p* = 0.0028). Between 2011 and 2014, a significant decline was observed, with an APC of −18.79% (95% CI: −24.52% to −5.55%, *p* = 0.0048). From 2014 to 2023, prices increased significantly, with an APC of 5.33% (95% CI: 2.28 to 14.45%, *p* = 0.0064). The AAPC for the entire period was 0.76% (95% CI: 0.22 to 1.48%, *p* = 0.0164), indicating a statistically significant overall increase in imipenem/cilastatin prices over the study period (Supplementary material).

### Meropenem

#### Meropenem utilization in Medicaid (1997–2023)

The quarterly trends in meropenem prescriptions in Medicaid from 1997 to 2023 show a steady increase over the years, interspersed with periods of fluctuation. The number of prescriptions began at 19 in the first quarter of 1997 and gradually increased, reaching over 1,400 prescriptions per quarter by the second quarter of 2002. A steady rise occurred between 2003 and 2008, with prescriptions peaking at 2,074 in the fourth quarter of 2008. From 2009 to 2014, prescription levels fluctuated between 2,000 and 4,000 per quarter, with a sharp peak of 9,088 prescriptions in the fourth quarter of 2014. Following this spike, there was a temporary decline in prescription numbers, which dropped to 4,281 in the fourth quarter of 2015. A steady growth phase resumed from 2016 onward, with prescriptions surpassing 7,000 per quarter by the second quarter of 2017. The most significant surge occurred in 2022, with prescriptions reaching a peak of 20,482 in the first quarter, the highest value recorded during the study period. This was followed by a sharp decline to 8,518 prescriptions in the fourth quarter of 2022. Prescription levels subsequently stabilized, fluctuating between 10,000 and 10,800 per quarter through 2023 (Figure 1).

The Joinpoint Regression analysis identified significant trends in prescriptions from 1997 to 2023. Between 1997 and 2016, the APC was 23.19% (95% CI: 18.07 to 55.02%, *p* = 0.0016). From 2016 to 2023, the APC was 2.66% (95% CI: −7.50 to 7.23%, *p* = 0.3643), indicating no statistically significant change during this period. The AAPC for the full range (1997–2023) was 17.29% (95% CI: 13.97 to 29.71%, *p* = 0.0016), reflecting a statistically significant overall increase in meropenem prescriptions (Supplementary material).

#### Meropenem reimbursement in Medicaid (1997–2023)

The quarterly reimbursement trends for meropenem in Medicaid from 1997 to 2023 demonstrate a steady upward trajectory with notable fluctuations. Reimbursements began at $16,454 in the first quarter of 1997 and gradually increased, reaching almost $500,000 by the second quarter of 2002. By the second quarter of 2004, reimbursements exceeded $1 million, reaching $1,039,803. From 2004 to 2006, reimbursements fluctuated within the range of $900,000 to $1.3 million per quarter. A consistent growth phase was observed between 2007 and 2014, with reimbursements peaking at $1.9 million in the fourth quarter of 2008. Between 2015 and 2019, periodic fluctuations occurred, with reimbursements ranging from $1.1 million to $2 million per quarter. A sharp peak was observed in the first quarter of 2021, when reimbursements reached $3.4 million, the highest recorded value in the study period. This was followed by a steep decline in 2022, with reimbursements dropping to approximately $1.4 million in the second quarter of that year. Reimbursements stabilized between $1.5 million and $2.1 million per quarter through the end of 2023 (Figure 2).

The Joinpoint Regression analysis for meropenem reimbursement from 1997 to 2023 showed a single segment with an APC of 3.53% (95% CI: 2.18 to 5.99%, *p* < 0.000001), indicating a statistically significant annual increase over the study period. The AAPC for the entire range was also 3.53% (95% CI: 2.18 to 5.99%, *p* < 0.000001), reflecting a consistent significant increase in reimbursement trends across the years (Supplementary material).

#### Meropenem price in Medicaid (1997–2023)

The quarterly proxy price trends for meropenem in Medicaid from 1997 to 2023 exhibit a gradual decline over time with distinct phases. Prices began at $866 per unit in the first quarter of 1997 and fluctuated within the $827 to $1,045 range until 1999. A gradual downward trend started in 2000, with prices dropping below $800 by the second quarter of 2000 and continuing to decrease to $513 by the fourth quarter of 2002. From 2003 to 2007, prices stabilized in the $500 to $800 range, with minor fluctuations. A slight increase occurred in 2007, with prices peaking at $1,048 in the third quarter, followed by fluctuations around $900 through 2009. A pronounced decline began in 2010, with prices dropping below $600 by the third quarter of 2010 and reaching $406 by the second quarter of 2012. Between 2013 and 2015, proxy prices continued to decline, falling below $300 in the second quarter of 2013 and reaching $165 in the third quarter of 2015. From 2016 to 2023, prices remained stable, fluctuating slightly around $200, with the lowest value recorded at $157 in the second quarter of 2023 (Figure 3).

The Joinpoint Regression analysis for meropenem price trends from 1997 to 2023 identified significant changes across five segments. From 1997 to 2003, the APC was −7.35% (95% CI: −8.74% to −6.24%, *p* < 0.000001), indicating a significant decline. Between 2003 and 2008, prices increased significantly, with an APC of 10.60% (95% CI: 8.92 to 12.38%, *p* < 0.000001). From 2008 to 2011, the APC was −15.83% (95% CI: −18.33% to −13.66%, *p* = 0.0112), showing a sharp decline. Between 2011 and 2014, the APC was −28.67% (95% CI: −32.19% to −24.07%, *p* = 0.0036), marking the steepest decline. From 2014 to 2023, the APC was −1.74% (95% CI: −4.32 to 2.07%, *p* = 0.297), with no statistically significant change. The AAPC for the entire period was −6.12% (95% CI: −6.80% to −5.63%, *p* < 0.000001), indicating a significant overall decline in meropenem prices over the study period (Supplementary material).

### Ertapenem

#### Ertapenem utilization in Medicaid (2002–2023)

The quarterly trends in ertapenem prescriptions in Medicaid from 2002 to 2023 demonstrate consistent growth over time with some fluctuations. Prescriptions began at 18 in the first quarter of 2002 and increased steadily, reaching approximately 3,000 prescriptions per quarter by the fourth quarter of 2005. Between 2006 and 2012, a significant growth phase was observed, with prescriptions rising from 2,446 in the first quarter of 2006 to 10,310 in the third quarter of 2012. The upward trend continued, with prescriptions surpassing 12,000 by 2014 and peaking at 15,038 in the fourth quarter of 2015. From 2016 to 2020, prescription levels fluctuated within the range of 16,000 to 20,000 per quarter. A dramatic increase occurred in 2021, with prescriptions reaching a peak of 38,794 in the second quarter of 2021, the highest value recorded during the study period. This was followed by a sharp decline, with prescriptions dropping to 16,800 by the fourth quarter of 2022. From 2022 onward, prescription levels stabilized, fluctuating between 20,000 and 23,000 per quarter through 2023 (Figure 1).

The Joinpoint Regression analysis for ertapenem prescriptions from 2002 to 2023 identified significant changes. Between 2002 and 2015, the APC was 19.05% (95% CI: 13.56 to 109.30%, *p* = 0.023), indicating a statistically significant increase. From 2015 to 2023, the APC was 7.79% (95% CI: −1.12 to 11.25%, *p* = 0.067), showing no statistically significant change. The AAPC for the entire period was 14.63% (95% CI: 11.60 to 27.88%, *p* = 0.021), reflecting a significant overall increase in ertapenem prescriptions over the study period (Supplementary material).

#### Ertapenem reimbursement in Medicaid (2002–2023)

The quarterly reimbursement trends for ertapenem in Medicaid from 2002 to 2023 show consistent growth with significant fluctuations in later years. Reimbursements started at $1,011 in the first quarter of 2002 and increased steadily, surpassing $500,000 per quarter by 2005. By the first quarter of 2009, reimbursements exceeded $1 million, reaching $1,020,827. Between 2010 and 2015, reimbursements continued to grow significantly, peaking at $4,502,308 in the fourth quarter of 2015. A substantial rise occurred in 2016, with reimbursements surpassing $6 million in the fourth quarter of 2016 and stabilizing within the range of $5 million to $6 million per quarter through 2020. A sharp spike was observed in 2022, with reimbursements peaking at $10,562,360 in the second quarter, marking the highest value recorded during the study period. This was followed by a rapid decline, with reimbursements dropping to $3,983,150 in the fourth quarter of 2022. From 2023 onward, reimbursements fluctuated between $4 million and $5.2 million per quarter (Figure 2).

The Joinpoint Regression analysis for ertapenem reimbursement from 2002 to 2023 identified significant trends. Between 2002 and 2017, the APC was 24.79% (95% CI: 20.00 to 37.69%, *p* < 0.000001), indicating a statistically significant increase. From 2017 to 2023, the APC was −0.16% (95% CI: −5.30 to 3.77%, *p* = 0.869), showing no statistically significant change. The AAPC for the entire period was 17.09% (95% CI: 14.40 to 23.90%, *p* < 0.000001), reflecting a significant overall increase in ertapenem reimbursement trends over the study period (Supplementary material).

#### Ertapenem price in Medicaid (2002–2023)

The quarterly proxy price trends for ertapenem in Medicaid from 2002 to 2023 show a distinct pattern of fluctuations followed by a decline in later years. Prices began at $56 per unit in the first quarter of 2002 and increased sharply to $238 by the second quarter of 2002. Between 2003 and 2008, prices fluctuated within the range of $200 to $280, with a peak of $279 in the third quarter of 2008. In 2009, proxy prices began to decline, falling to $193 in the first quarter, but stabilized around $200 and $270 through 2012. A gradual increase followed, with prices reaching $280 by the first quarter of 2015 and peaking at $333 in the fourth quarter of 2016. From 2017 to 2018, prices rose significantly, reaching a high of $453 in the first quarter of 2018 before beginning a downward trend. By 2020, prices stabilized in the $290 to $310 range, followed by a steady decline to $235 by the first quarter of 2023. Prices showed slight recovery in the later quarters of 2023, ending at $230 in the fourth quarter (Figure 3).

The Joinpoint Regression analysis for ertapenem price trends from 2002 to 2023 identified significant changes across three segments. From 2002 to 2013, the APC was 0.62% (95% CI: −2.26 to 2.29%, *p* = 0.615), indicating no statistically significant change. Between 2013 and 2018, the APC was 11.89% (95% CI: 7.77 to 19.74%, *p* < 0.000001), showing a significant increase. From 2018 to 2023, the APC was −11.39% (95% CI: −15.22% to −7.83%, *p* < 0.000001), reflecting a significant decline. The AAPC for the entire period was 0.12% (95% CI: −0.71 to 0.88%, *p* = 0.806), indicating no significant overall change in ertapenem prices over the study period (Supplementary material).

### Doripenem

#### Doripenem utilization in Medicaid (2008–2017)

The quarterly trends in doripenem prescriptions in Medicaid from 2008 to 2017 exhibit a rapid rise, followed by a gradual decline. Prescriptions started at minimal levels, with 4 prescriptions in the first quarter of 2008, and increased steadily, surpassing 400 prescriptions per quarter by the third quarter of 2009. A sharp growth phase occurred from 2010 to the second quarter of 2011, where prescriptions peaked at 859 in the second quarter of 2011, marking the highest recorded value during the study period. Subsequently, prescription levels began to decline steadily, falling to 591 in the first quarter of 2012 and dropping further to 264 by the third quarter of 2012. Between 2013 and 2014, prescription levels continued to decrease, with notable reductions to 91 prescriptions by the third quarter of 2014 and 52 prescriptions by the fourth quarter. From 2015 to 2017, the decline persisted, with quarterly prescriptions fluctuating between 40 and 141. By the fourth quarter of 2017, prescriptions had dropped to just 11, reflecting the lowest recorded value during the study period (Figure 1).

The Joinpoint Regression analysis for doripenem prescriptions from 2008 to 2017 identified significant trends across two segments. From 2008 to 2011, the APC was 49.31% (95% CI: 27.90 to 90.46%, *p* < 0.000001), indicating a significant increase. From 2011 to 2017, the APC was −39.24% (95% CI: −52.08% to −35.15%, *p* < 0.000001), reflecting a significant decline. The AAPC for the entire period was −18.00% (95% CI: −29.20% to −13.42%, *p* = 0.0012), showing an overall significant decrease in doripenem prescriptions over the study period (Supplementary material).

#### Doripenem reimbursement in Medicaid (2008–2017)

The quarterly reimbursement trends for doripenem in Medicaid from 2008 to 2017 show an initial sharp increase, followed by significant fluctuations and a prolonged decline. Reimbursements began at $5,211 in the first quarter of 2008 and grew steadily, surpassing $88,000 by the fourth quarter of 2008. A substantial rise occurred in 2009, with reimbursements peaking at $540,522 in the fourth quarter. In 2010, reimbursements exhibited variability, fluctuating between $164,046 and $250,896 per quarter. A secondary peak was observed in the second quarter of 2011, with reimbursements reaching $337,422, the highest value recorded during the study period. Following this peak, a gradual decline began, with reimbursements dropping to $213,445 by the first quarter of 2012. Between 2013 and 2014, reimbursements declined significantly, falling to $58,980 by the first quarter of 2014 and further dropping to $17,826 by the fourth quarter. From 2015 to 2017, reimbursements showed sporadic increases, with minor peaks such as $74,302 in the third quarter of 2015. By the fourth quarter of 2017, reimbursements had decreased to $3,622, marking the lowest value during the analysis period (Figure 2).

The Joinpoint Regression analysis for doripenem reimbursement from 2008 to 2017 identified significant trends across two segments. From 2008 to 2011, the APC was 21.54% (95% CI: 3.23 to 44.42%, *p* = 0.013), indicating a statistically significant increase. Between 2011 and 2017, the APC was −37.03% (95% CI: −53.26% to −32.94%, *p* < 0.000001), reflecting a significant decline. The AAPC for the entire period was −21.60% (95% CI: −33.96% to −18.05%, *p* < 0.000001), showing an overall significant decrease in doripenem reimbursement over the study period (Supplementary material).

#### Doripenem price in Medicaid (2008–2017)

The quarterly proxy price trends for doripenem in Medicaid from 2008 to 2017 demonstrate notable variability. Prices began at $1,303 per unit in the first quarter of 2008 and declined sharply to $499 per unit by the third quarter of the same year. By the fourth quarter of 2009, proxy prices peaked again at $1,302, indicating a dramatic short-term increase. From 2010 to 2012, prices exhibited a general downward trend, fluctuating between $363 and $526 per unit. A moderate stabilization was observed during this period, with occasional increases such as $526 in the third quarter of 2012. Between 2013 and 2014, prices continued to decline, reaching a low of $265 in the third quarter of 2014. However, an upward shift occurred from 2015, with prices peaking at $571 in the second quarter of 2015. From 2016 onward, prices fluctuated significantly, with a low of $223 in the third quarter of 2016 and a subsequent sharp rise to $778 in the second quarter of 2017. By the fourth quarter of 2017, prices had decreased again to $329, reflecting ongoing volatility (Figure 3).

The Joinpoint Regression analysis for doripenem prices from 2008 to 2017 identified two distinct trends. From 2008 to 2010, the APC was −33.83% (95% CI: −44.54% to −17.66%, *p* < 0.000001), indicating a statistically significant decline. Between 2010 and 2017, the APC was 1.73% (95% CI: −3.48 to 14.60%, *p* = 0.436), showing no statistically significant change. The AAPC for the entire period was −7.54% (95% CI: −10.94% to −4.58%, *p* < 0.000001), reflecting an overall significant decrease in doripenem prices over the study period (Supplementary material).

### Comparing carbapenems prescriptions

The trend analysis reveals notable shifts in the utilization of carbapenems in Medicaid from 1991 to 2023. Imipenem/cilastatin initially dominated prescriptions, peaking in 2005 at 22,883 prescriptions, followed by a continuous decline to 2,331 in 2023, indicating a significant reduction in its clinical preference over time. Meropenem showed a steady increase in use since its introduction, reaching a peak of 53,355 prescriptions in 2016. Its utilization has since stabilized, with 41,613 prescriptions recorded in 2023. Ertapenem, introduced in 2002, demonstrated the most substantial growth among the carbapenems, surpassing both Imipenem/Cilastatin and meropenem by 2015. By 2022, it reached its peak utilization at 116,988 prescriptions, affirming its emergence as the most widely used carbapenem. A slight decline was observed in 2023, with 87,956 prescriptions. In contrast, doripenem, introduced in 2008, experienced minimal adoption, with peak prescriptions of only 2,954 in 2011, followed by a steady decline to negligible levels in subsequent years.

### Comparing carbapenems reimbursements

The reimbursement trends for carbapenems in Medicaid from 1991 to 2023 highlight distinct patterns across the four agents. Imipenem/cilastatin showed a peak reimbursement of $7,555,204 in 2005, followed by a steady decline to $839,581 in 2023, reflecting its reduced clinical preference over time. Meropenem experienced a gradual increase in reimbursements, peaking at $10,948,523 in 2016, with fluctuations in subsequent years and a reimbursement of $7,198,477 in 2023, indicating sustained but moderated use. Ertapenem, introduced in 2002, exhibited the most substantial growth, surpassing all other carbapenems by 2016 and peaking at $30,749,447 in 2022. While reimbursements declined to $19,530,233 in 2023, ertapenem remains the most reimbursed carbapenem, highlighting its widespread clinical adoption. Conversely, doripenem, introduced in 2008, consistently showed the lowest reimbursements, peaking at $1,204,343 in 2011 and declining to negligible levels in recent years, reflecting its limited market impact.

### Comparing carbapenems price

The proxy price trends for carbapenems in Medicaid from 1991 to 2023 reveal significant variation across the four agents. Imipenem/Cilastatin displayed relatively stable pricing until 2014, with minor fluctuations and a slight decrease from $238 in 1991 to $190 in 2014. However, prices increased again after 2015, peaking at $363 in 2023. Meropenem started with high initial pricing upon introduction, reaching $939 in 1997, followed by a gradual and consistent decline to $168 in 2022, indicating cost reduction over time. Ertapenem, introduced in 2002, exhibited moderate pricing stability initially, ranging between $179 and $251 until 2008, followed by a sharp increase peaking at $415 in 2018. A subsequent decline brought the price down to $222 in 2023. Doripenem, introduced in 2008, showed high initial prices, peaking at $915 in 2008. Prices then declined sharply, stabilizing around $258 in 2016, with notable fluctuations afterward, reaching $486 in 2017 and $222 in 2023.

### Carbapenems prescriptions market share

**Figure 4:**
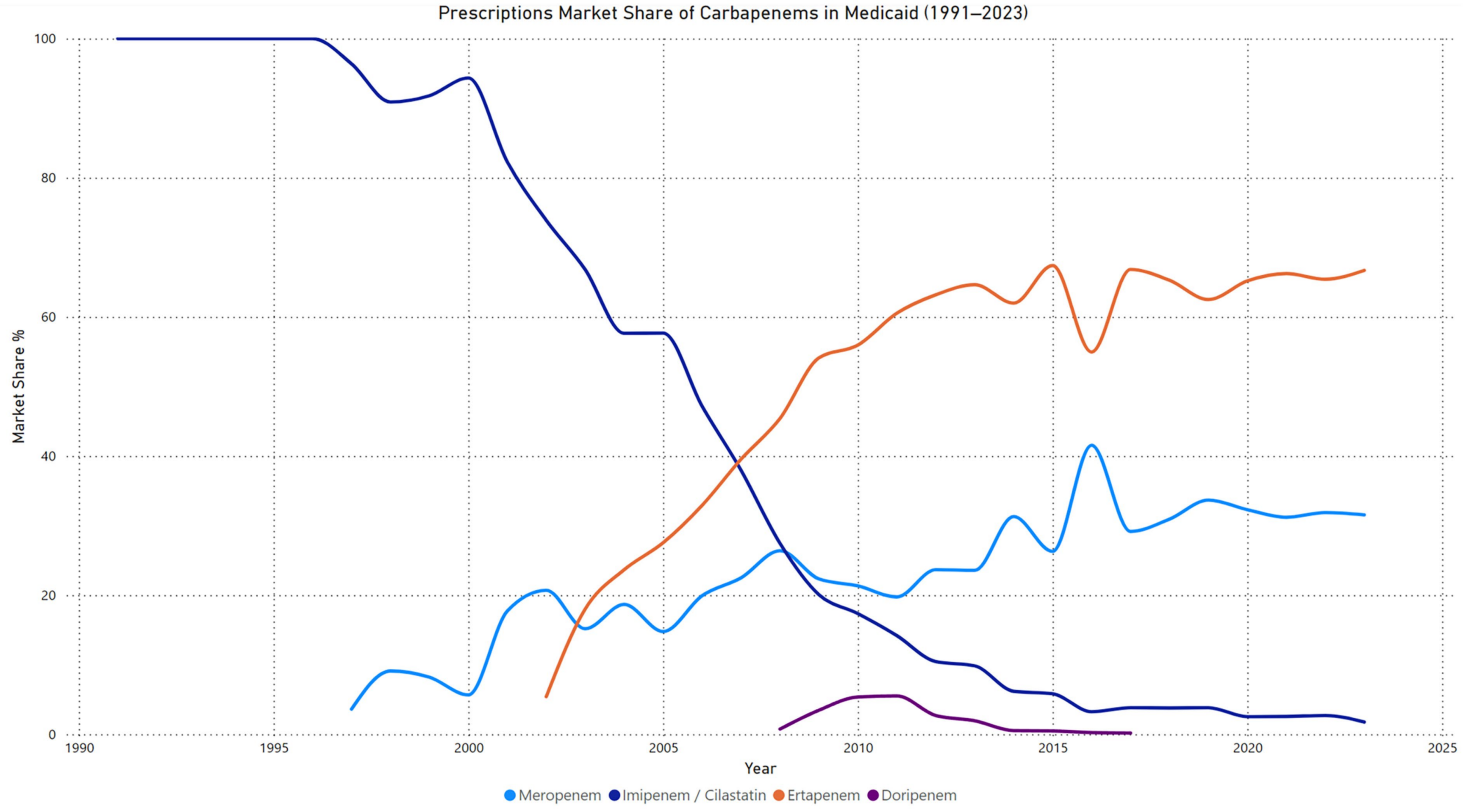
Carbapenems prescription market share price in Medicaid.

The market share analysis for carbapenems in Medicaid from 1991 to 2023 highlights distinct trends among the four agents. Imipenem/cilastatin maintained 100% market share until 1996 but experienced a consistent decline thereafter, reaching just 1.8% by 2023. This reflects a significant shift away from its dominance as newer agents gained traction. Meropenem entered the market in 1997 and steadily increased its share, peaking at 41.5% in 2016. Its market share stabilized around 31.5% in 2023, suggesting continued clinical reliance but moderated growth. Ertapenem, introduced in 2002, quickly gained market dominance, surpassing all other carbapenems by 2007. It consistently held the highest market share from 2008 onward, peaking at 67.4% in 2015 and maintaining 66.7% in 2023, underscoring its preference in clinical practice. In contrast, doripenem, introduced in 2008, had minimal impact, with its market share peaking at 5.5% in 2011 before declining to 0% by 2018 (Figure 4).

### Carbapenems reimbursements market share

**Figure 5:**
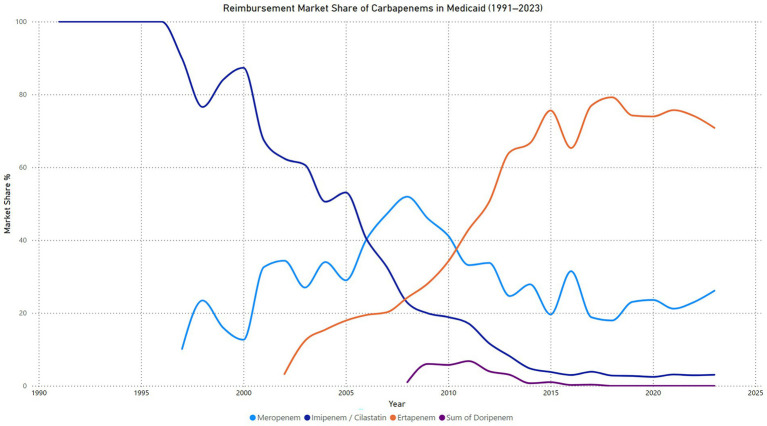
Carbapenems reimbursement market share price in Medicaid.

The reimbursement market share for carbapenems in Medicaid from 1991 to 2023 demonstrates significant shifts over time. Imipenem/cilastatin held 100% of the reimbursement market until 1996, after which its share began to decline steadily, reaching just 3.0% in 2023. This trend reflects its decreasing utilization and preference as newer carbapenems entered the market. Meropenem emerged in 1997 and experienced a gradual increase in market share, peaking at 51.9% in 2008. Although its share fluctuated in subsequent years, it maintained a consistent presence, with 26.1% in 2023. Ertapenem, introduced in 2002, quickly rose to dominance, surpassing all other carbapenems by 2013 and reaching its peak market share of 79.2% in 2018. Despite a slight decline, ertapenem remained the leading carbapenem in reimbursement terms, holding 70.8% of the market in 2023. In contrast, doripenem, introduced in 2008, showed limited adoption, with its highest market share of 6.8% in 2011. By 2018, its reimbursement share dropped to 0%, indicating minimal impact on the market (Figure 5).

## Discussion

This study examined the longitudinal trends in the utilization, reimbursement, and pricing of carbapenem antibiotics within the Medicaid program from 1991 to 2023. The findings reveal significant shifts in prescribing patterns, cost dynamics, and market shares among the four carbapenems: imipenem/cilastatin, meropenem, ertapenem, and doripenem.

Imipenem/cilastatin, the first carbapenem introduced, initially dominated the market, maintaining a 100% market share until 1996. However, its utilization and reimbursement steadily declined following the introduction of newer carbapenems, particularly meropenem and ertapenem. This trend aligns with the broader adoption of alternative agents and evolving clinical guidelines favoring more targeted therapies to mitigate antimicrobial resistance (11–16). The decline in Imipenem/cilastatin prescriptions post-2005 can be attributed to safety concerns and the introduction of newer carbapenems with more favorable dosing profiles. Studies have indicated that imipenem, especially at higher doses, carries a risk of neurotoxicity, including seizures, particularly in patients with renal impairment (17). Comparative studies have demonstrated that meropenem is associated with a lower incidence of Adverse Effects (AEs) compared to imipenem/cilastatin. Specifically, research indicates that nausea and vomiting occur less frequently with meropenem. For instance, a review in Mayo Clinic Proceedings reported that in comparative trials, meropenem had lower rates of serious AEs compared to imipenem/cilastatin (18, 19). Additionally, a study published in Critical Care found no drug-related nausea and vomiting in patients treated with meropenem, whereas one drug-related seizure was reported in the imipenem/cilastatin group. These findings suggest that meropenem has a more favorable safety profile, particularly concerning gastrointestinal and neurological adverse effects (20, 21). Furthermore, the therapeutic margin is narrower with Imipenem/cilastatin, limiting its dosing flexibility. This limitation has led clinicians to prefer newer carbapenems that offer more straightforward dosing regimens and improved safety profiles (17). These factors have collectively influenced a shift in clinical practice, favoring the use of newer carbapenems over Imipenem/cilastatin in the years following 2005. Imipenem/cilastatin was first approved in 1985, meropenem in 1996, ertapenem in 2001, and doripenem in 2007. These approval dates align with the observed shifts in market share, particularly the initial dominance of imipenem/cilastatin and the rapid rise of ertapenem and meropenem in subsequent years.

Meropenem exhibited a consistent upward trajectory in both prescriptions and reimbursements following its introduction in 1997, peaking in 2016. Its increased use reflects its broad-spectrum activity, improved safety profile, and clinical effectiveness, particularly in severe Gram-negative infections (22–24). Studies have demonstrated that meropenem has a favorable safety profile, with a lower incidence of adverse effects compared to other carbapenems. This has contributed to its increased utilization in clinical practice (25). However, its market share stabilized after 2016, possibly due to the impact of antimicrobial stewardship programs aimed at controlling carbapenem overuse to prevent resistance (26, 27). Studies have shown that such stewardship initiatives can significantly influence prescribing behaviors and reduce the incidence of carbapenem-resistant organisms (28, 29).

Ertapenem, introduced in 2002, demonstrated the most substantial growth in both utilization and reimbursement. By 2015, it had surpassed both imipenem/cilastatin and meropenem, becoming the most widely prescribed and reimbursed carbapenem. Ertapenem differs from other carbapenems in its narrower spectrum and once-daily dosing, making it particularly suitable for community-acquired infections and outpatient parenteral antibiotic therapy. These characteristics likely contributed to its increased adoption and eventual surpassing of imipenem and meropenem in Medicaid prescriptions (30–32). However, a decline in utilization and reimbursement after 2021 may indicate a shift towards more targeted therapies or the influence of stewardship programs limiting broad-spectrum antibiotic use (33–35).

Doripenem, introduced in 2008, failed to gain significant market share due to clinical concerns over its safety, particularly in ventilator-associated pneumonia (VAP), and limited clinical advantages. In 2014, the U.S. FDA issued a warning about an increased risk of death and lower clinical cure rates when doripenem was used to treat VAP, compared to imipenem and cilastatin (36). This led to a decline in its utilization and eventual market withdrawal. The European Medicines Agency (EMA) also noted that the marketing authorization holder voluntarily withdrew doripenem from the European market for commercial reasons (37). These developments underscore doripenem’s limited clinical utility and market impact.

Pricing trends varied across carbapenems. Imipenem/cilastatin and ertapenem exhibited moderate price increases in recent years, while meropenem experienced a steady decline, likely due to the introduction of generics and market competition (38). Doripenem’s pricing remained highly volatile, reflecting its declining and limited market presence. The observed declines in reimbursement for certain carbapenems may partially reflect the entry of generic formulations, which typically reduce per-unit costs through increased market competition. Although the Medicaid dataset does not distinguish between branded and generic versions, the timing of generic availability likely played a role in the downward trend.

The reduction in carbapenem prescriptions observed after 2021 may reflect enhanced antimicrobial stewardship efforts aimed at curbing overuse of broad-spectrum antibiotics. Additionally, policy changes promoting the use of narrower-spectrum agents and growing awareness of resistance risks have likely influenced prescribing patterns.

Overall, the substantial rise in carbapenem utilization and expenditures from 2015 to 2020 highlights growing clinical reliance on these agents, potentially driven by the increasing burden of multidrug-resistant organisms (23, 39). However, the decline in both prescriptions and reimbursements after 2021 suggests that antimicrobial stewardship efforts, policy changes, and the availability of novel therapies may be influencing prescribing practices (26, 33). These findings underscore the need for continuous monitoring of antibiotic utilization to balance effective infection management with the containment of antimicrobial resistance and healthcare costs. Further research is warranted to explore the factors driving these trends and to evaluate the impact of stewardship initiatives on carbapenem prescribing.

The dynamic trends in carbapenem utilization emphasize the importance of ongoing surveillance to ensure appropriate use. Continuous monitoring enables healthcare systems to adapt to emerging resistance patterns and to implement timely interventions that balance the necessity of effective infection management with the imperative to mitigate antimicrobial resistance and control healthcare expenditures. To fully understand the drivers behind these prescribing trends, additional research is essential. Investigating the specific factors influencing clinicians’ decisions, evaluating the long-term outcomes of stewardship initiatives, and assessing the impact of new therapeutic options will provide valuable insights. Such studies are crucial for developing strategies that promote the judicious use of carbapenems and other critical antibiotics in the face of evolving resistance challenges.

## Conclusion

This study highlights the evolving trends in carbapenem utilization, reimbursement, and pricing within the Medicaid program from 1991 to 2023. The findings demonstrate the impact of clinical practices, antimicrobial stewardship efforts, and policy changes on prescribing patterns and market dynamics. These results emphasize the need for continuous monitoring and targeted interventions to optimize carbapenem use, address emerging resistance challenges, and ensure cost-effective infection management in Medicaid populations. Further research should focus on evaluating the long-term effects of stewardship programs and novel therapeutic options on carbapenem utilization and outcomes.

## Data Availability

The datasets presented in this study can be found in online repositories. The names of the repository/repositories and accession number(s) can be found at: Centers for Medicare & Medicaid Services (CMS) https://data.cms.gov/.
