# Supplementary material for: Trends in carbapenem antibiotics utilization, costs, and market dynamics in Medicaid: a retrospective analysis from 1991 to 2023
Source: Front Med (Lausanne). 2025 Aug 7;12:1589981. doi: 10.3389/fmed.2025.1589981 (PMC12367653; doi:10.3389/fmed.2025.1589981)

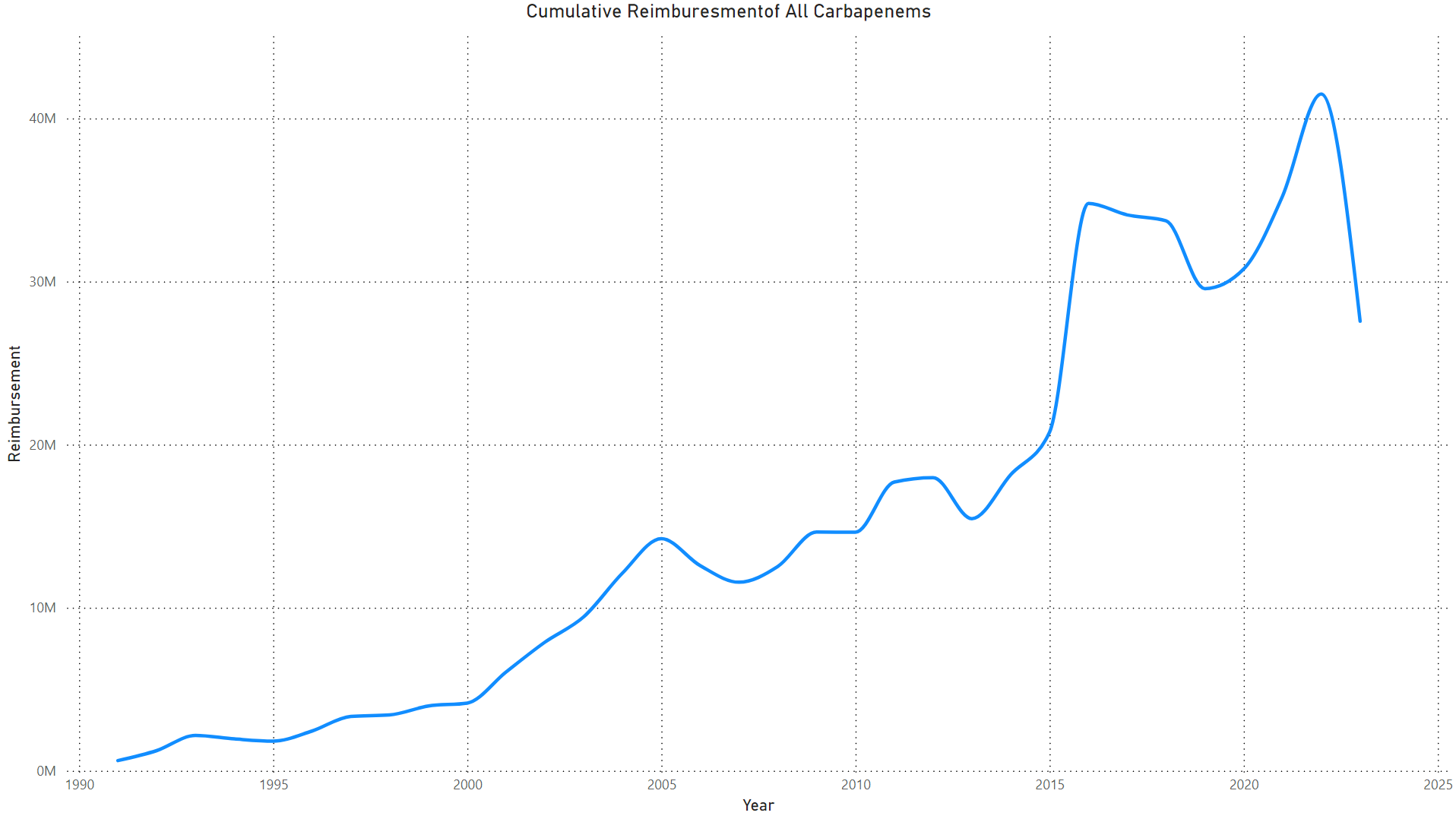

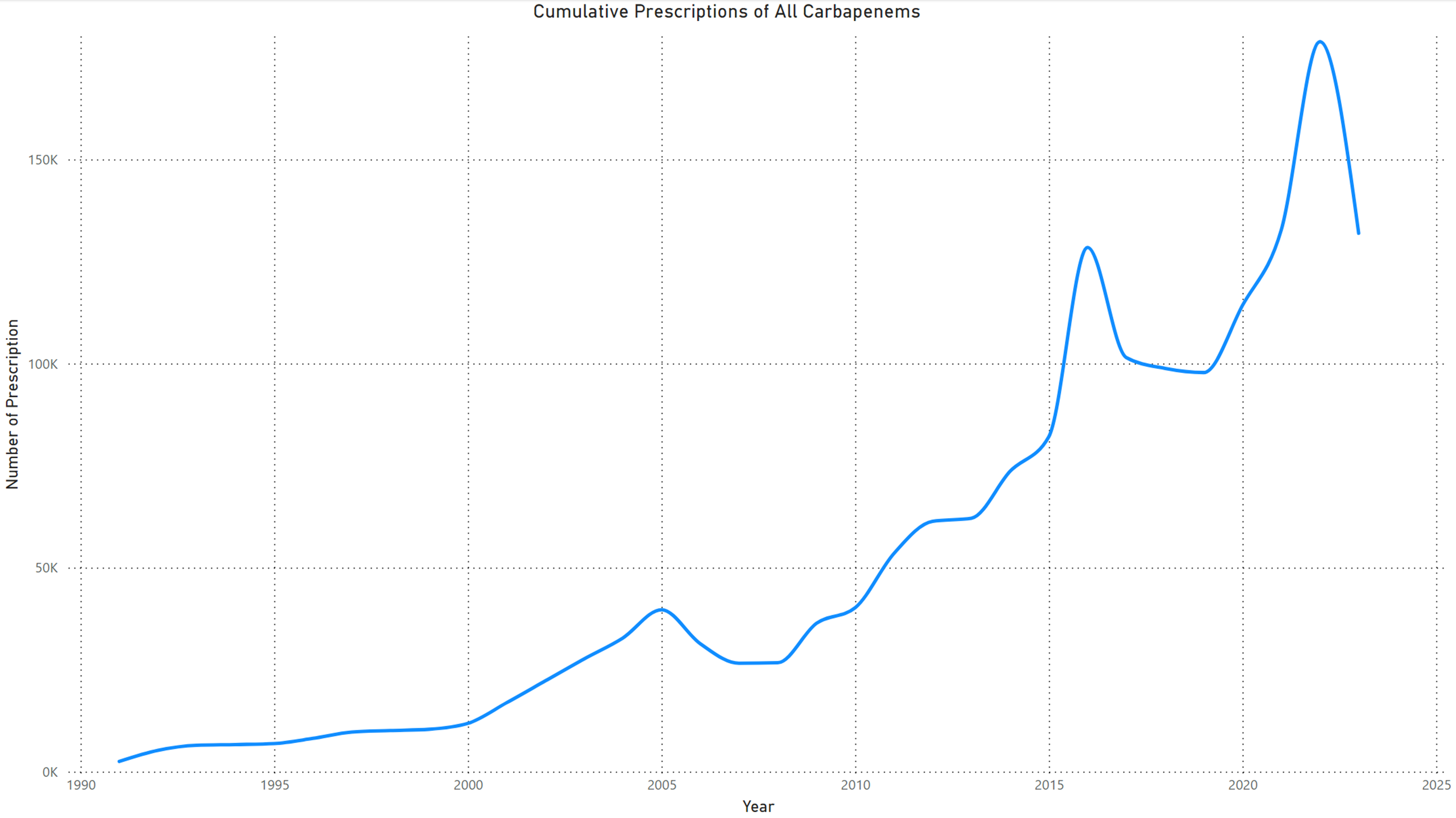
Figure S1: Cumulative utilization of Imipenem/Cilastatin, Meropenem, Ertapenem, and Doripenem in Medicaid from 1991 to 2023

Figure S2: Cumulative reimbursement of Imipenem/Cilastatin, Meropenem, Ertapenem, and Doripenem in Medicaid from 1991 to 2023

Figure S3: Join point regression of Imipenem/Cilastatin utilization in Medicaid.


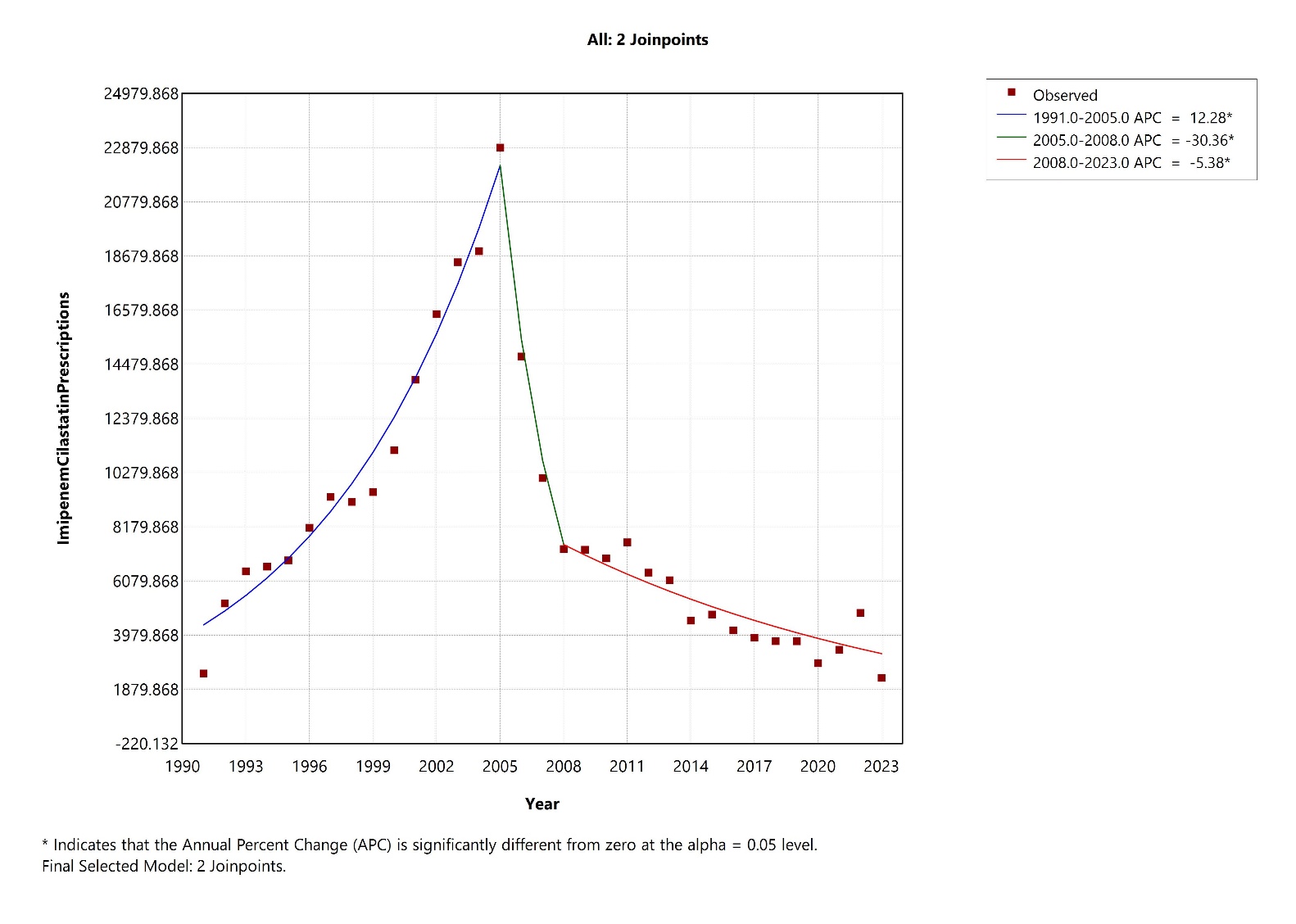

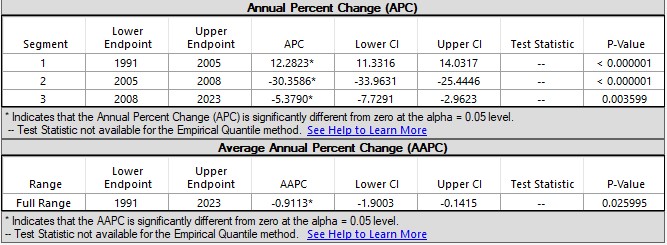


Figure S4: Join point regression of Imipenem/Cilastatin reimbursement in Medicaid


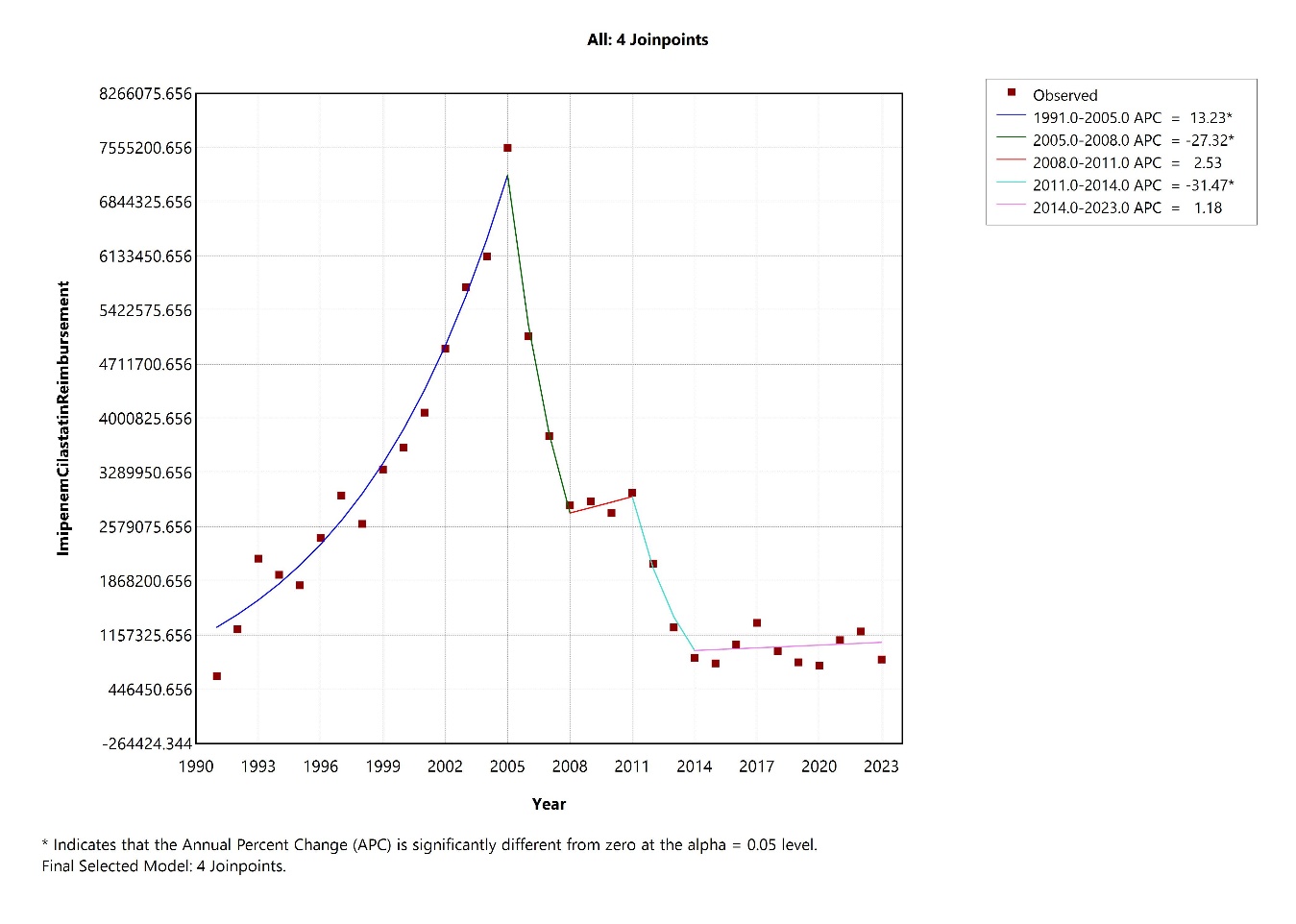

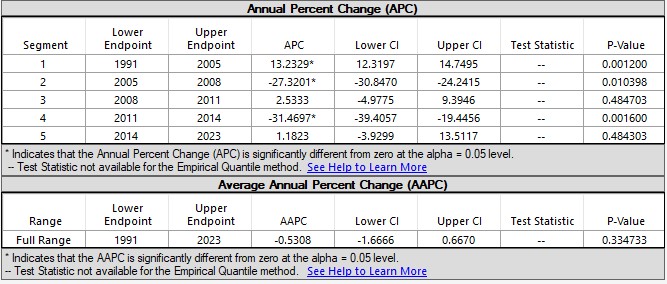

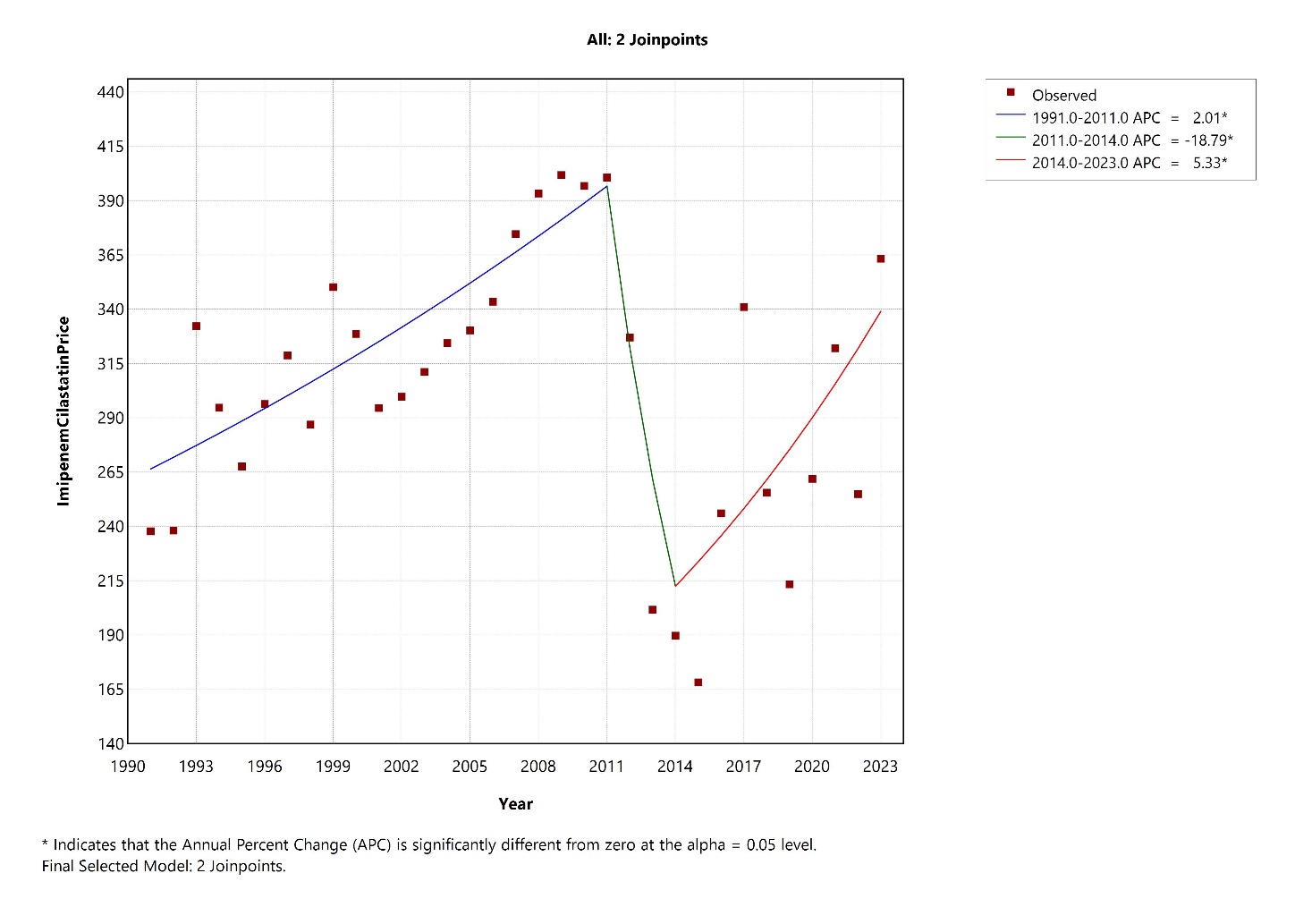

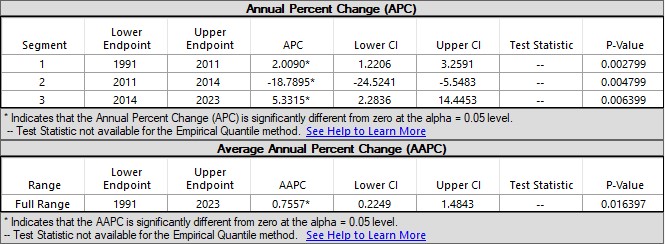


Figure S5: Join point regression of Imipenem/Cilastatin proxy price in Medicaid


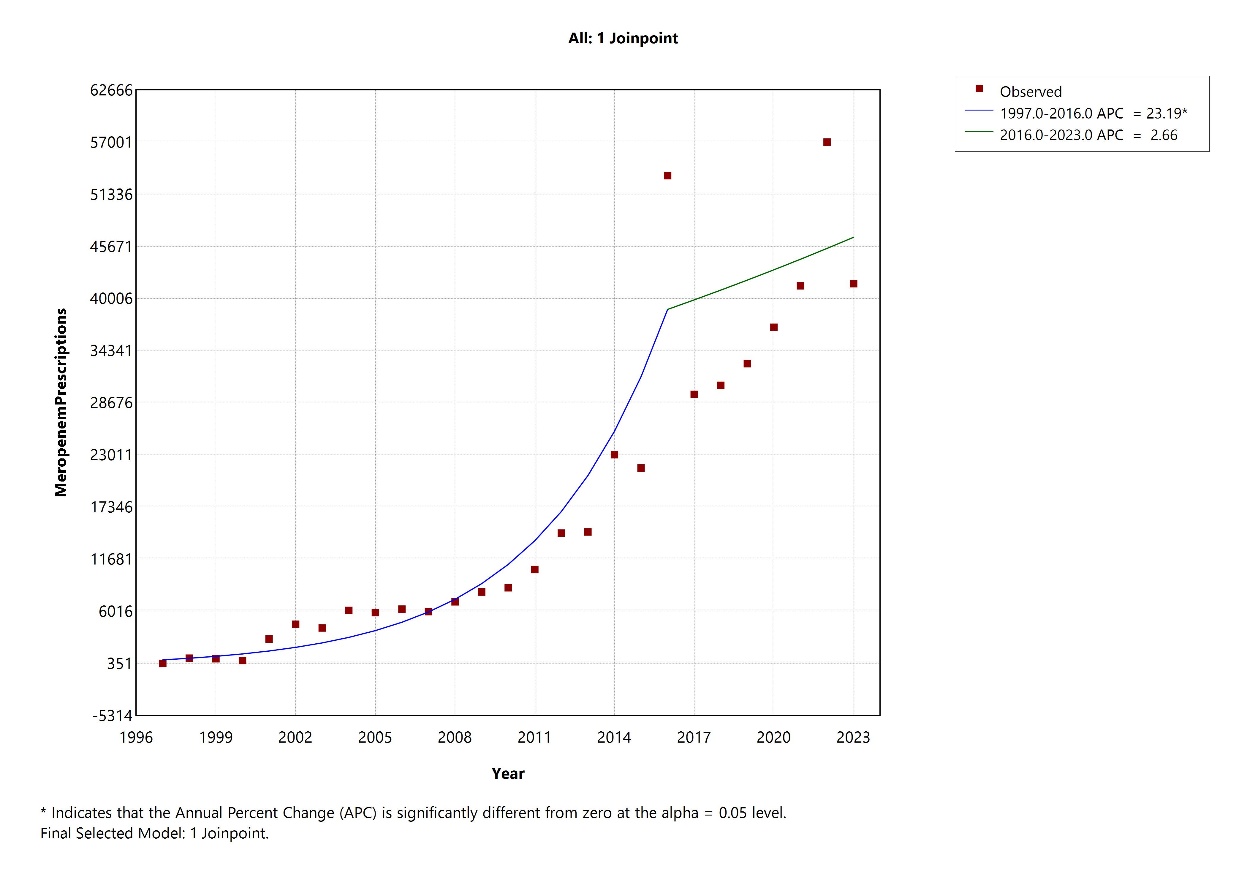

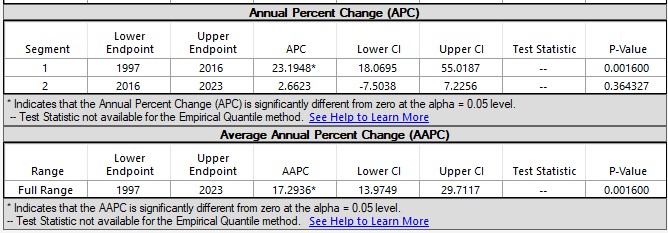


Figure S6: Join point regression of Meropenem utilization in Medicaid

Figure S7: Join point regression of Meropenem reimbursement in Medicaid


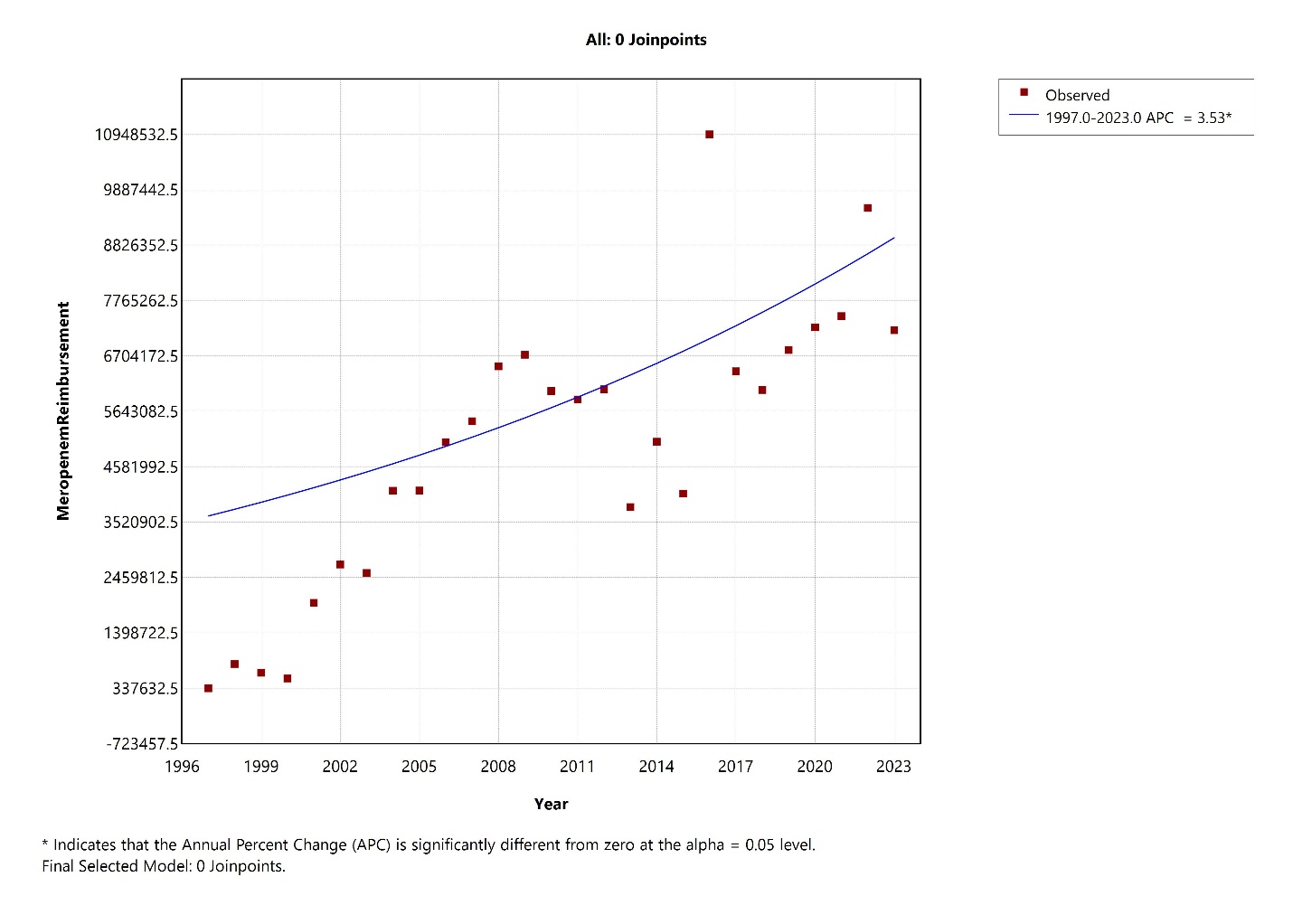

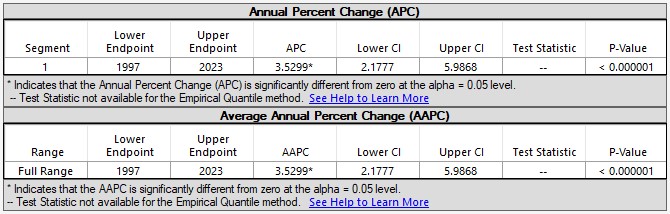


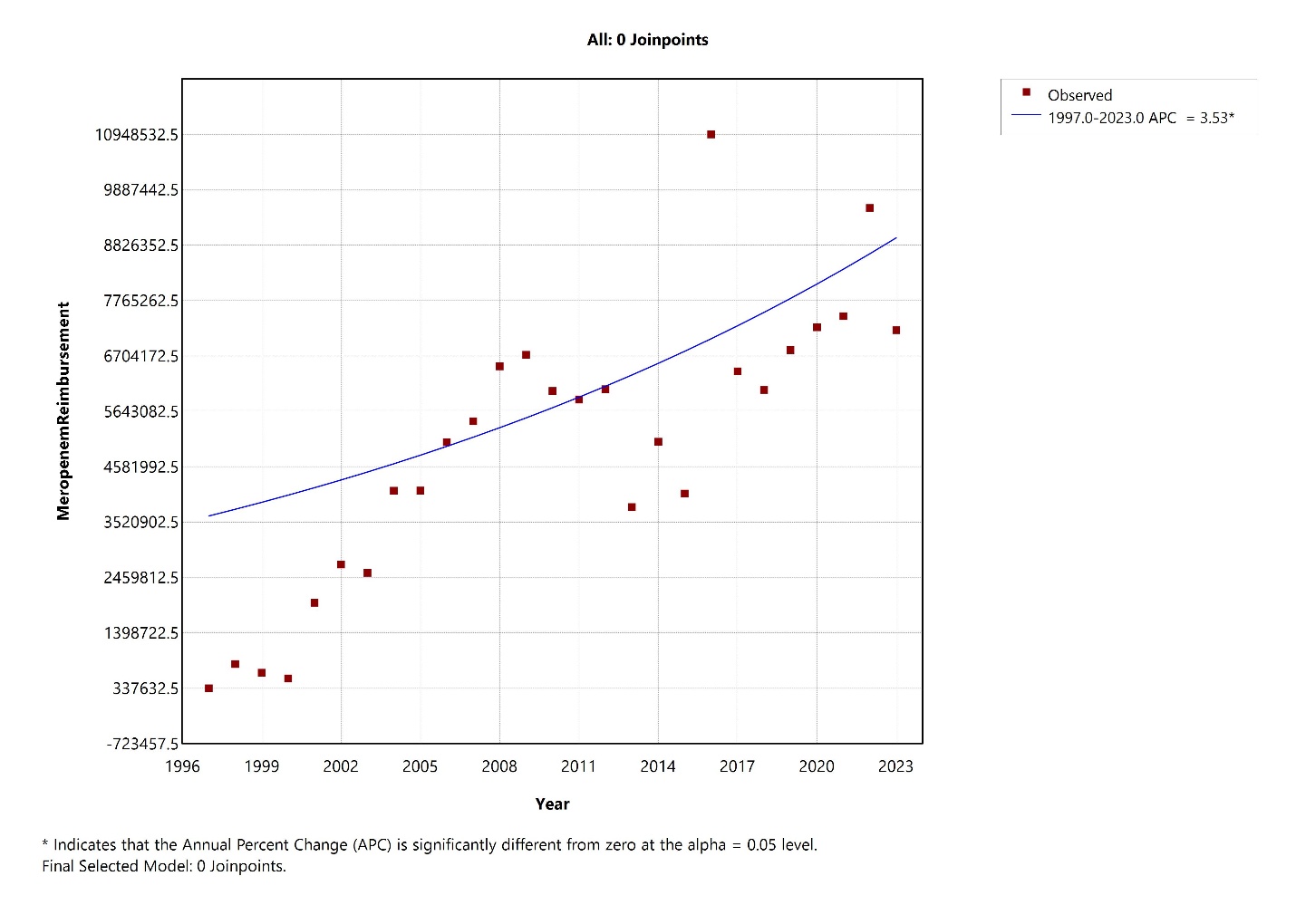


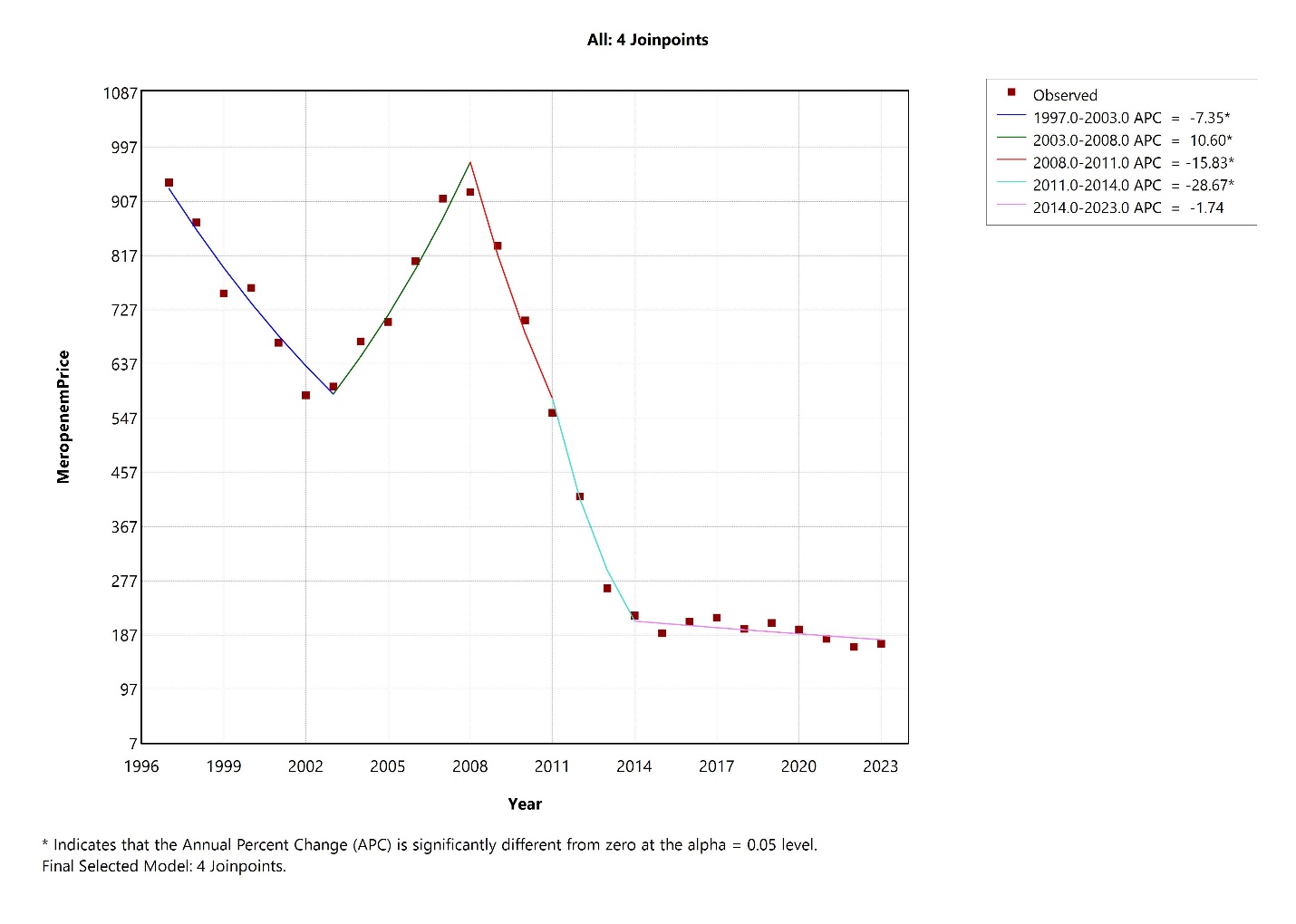

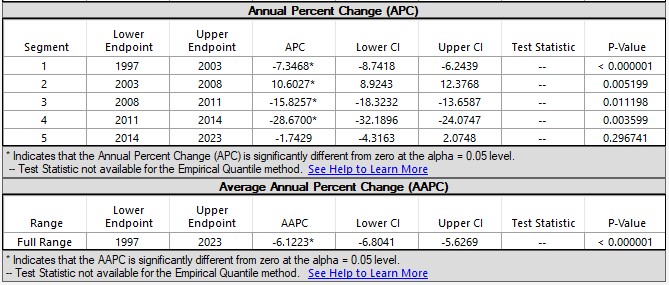


Figure S8: Join point regression of Meropenem proxy price in Medicaid

Figure S9: Join point regression of Ertapenem utilization in Medicaid


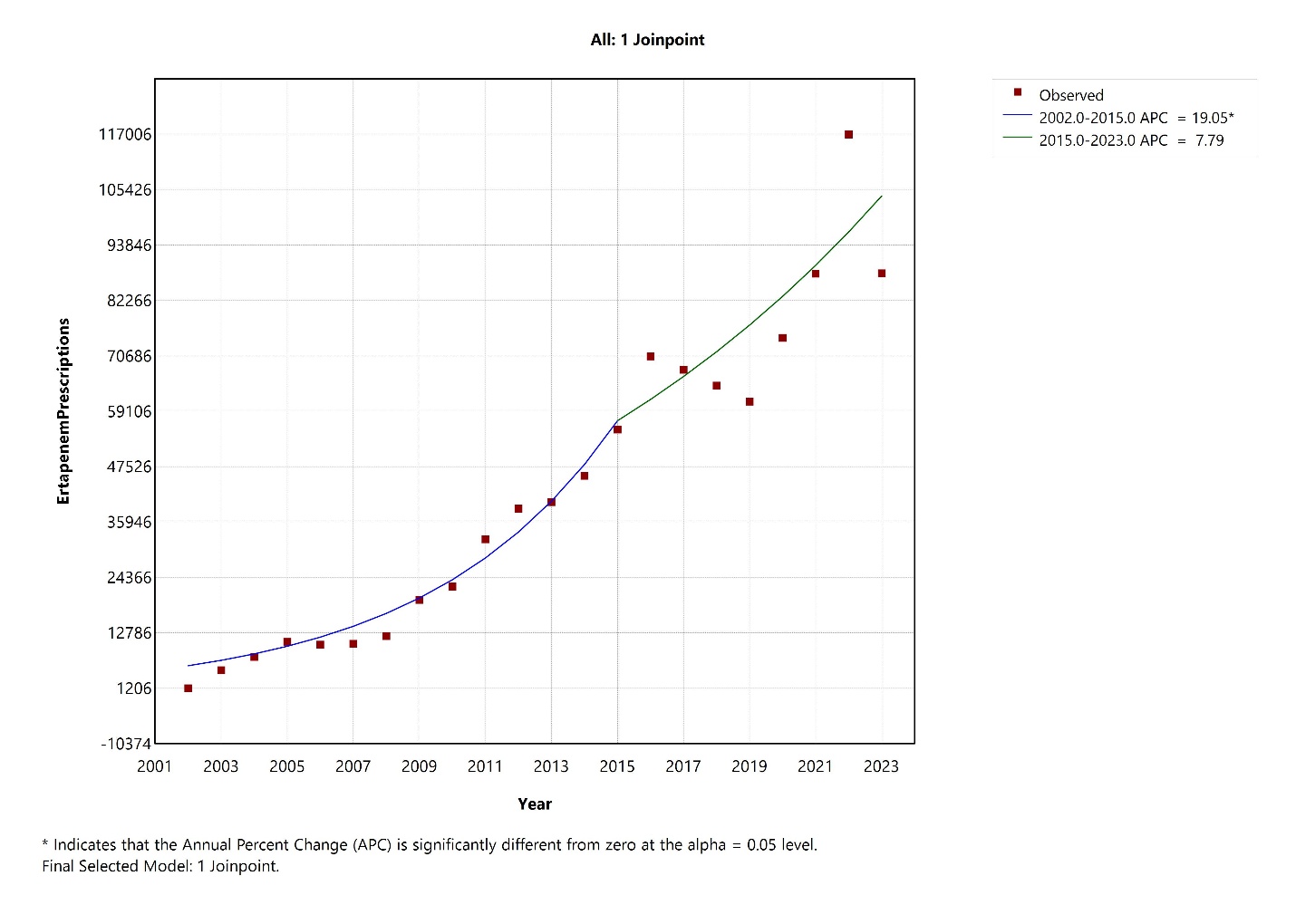

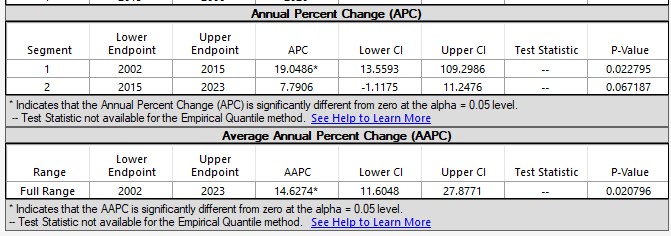


Figure S10: Join point regression of Ertapenem reimbursement in Medicaid


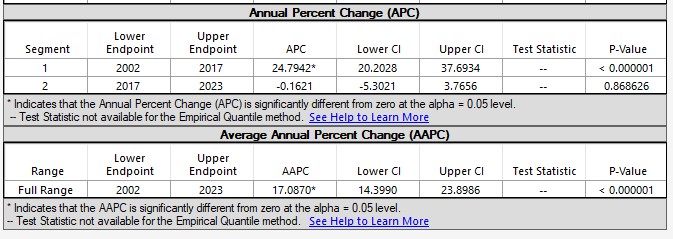

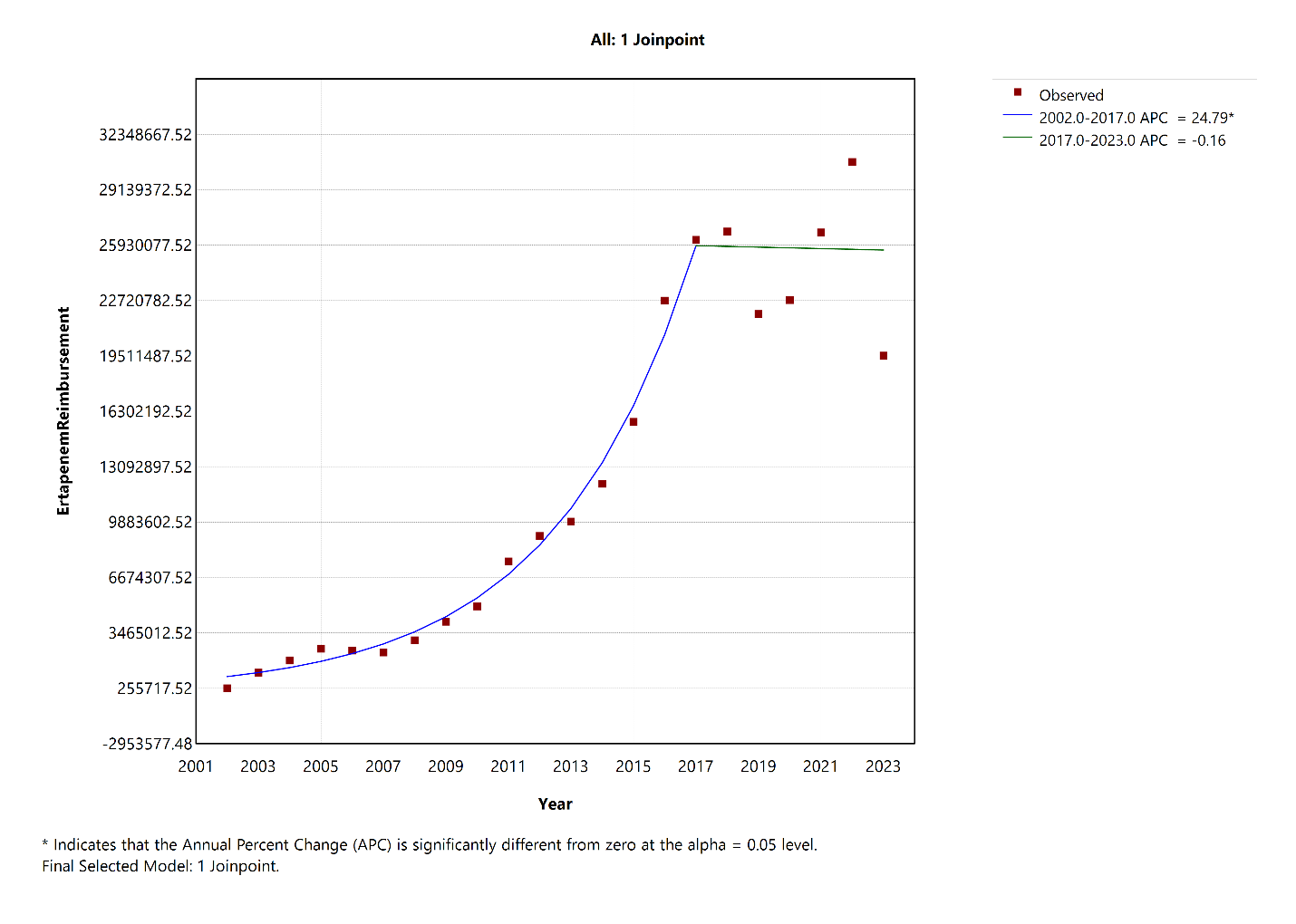


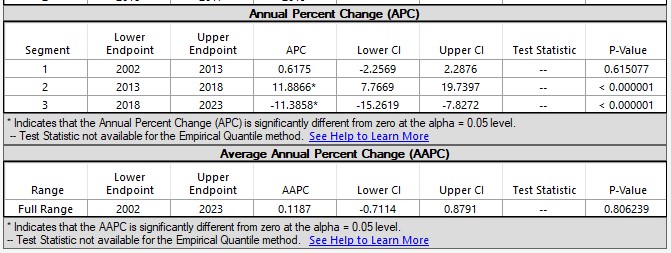

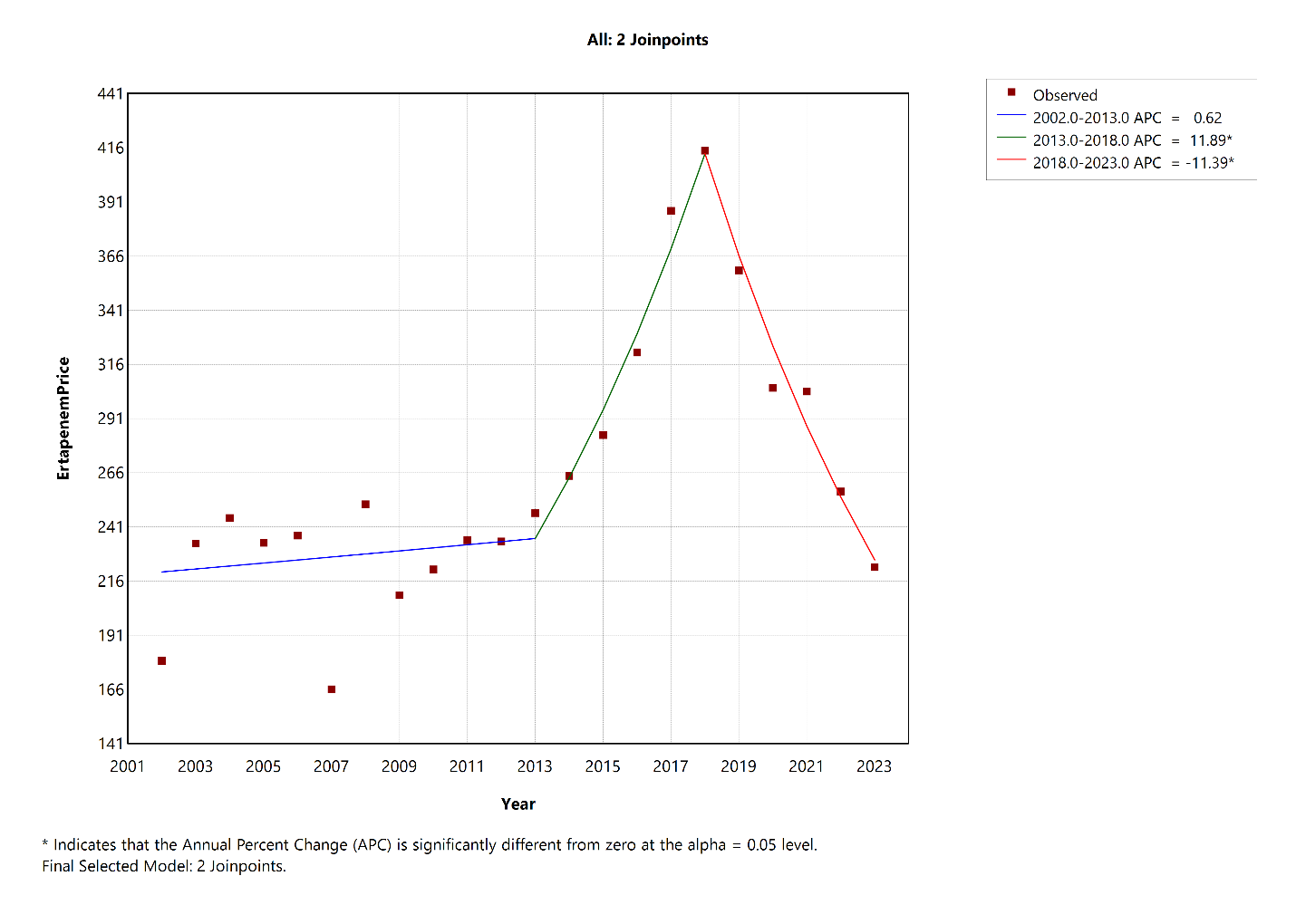


Figure S11: Join point regression of Ertapenem proxy price in Medicaid

Figure S12: Join point regression of Doripenem utilization in Medicaid


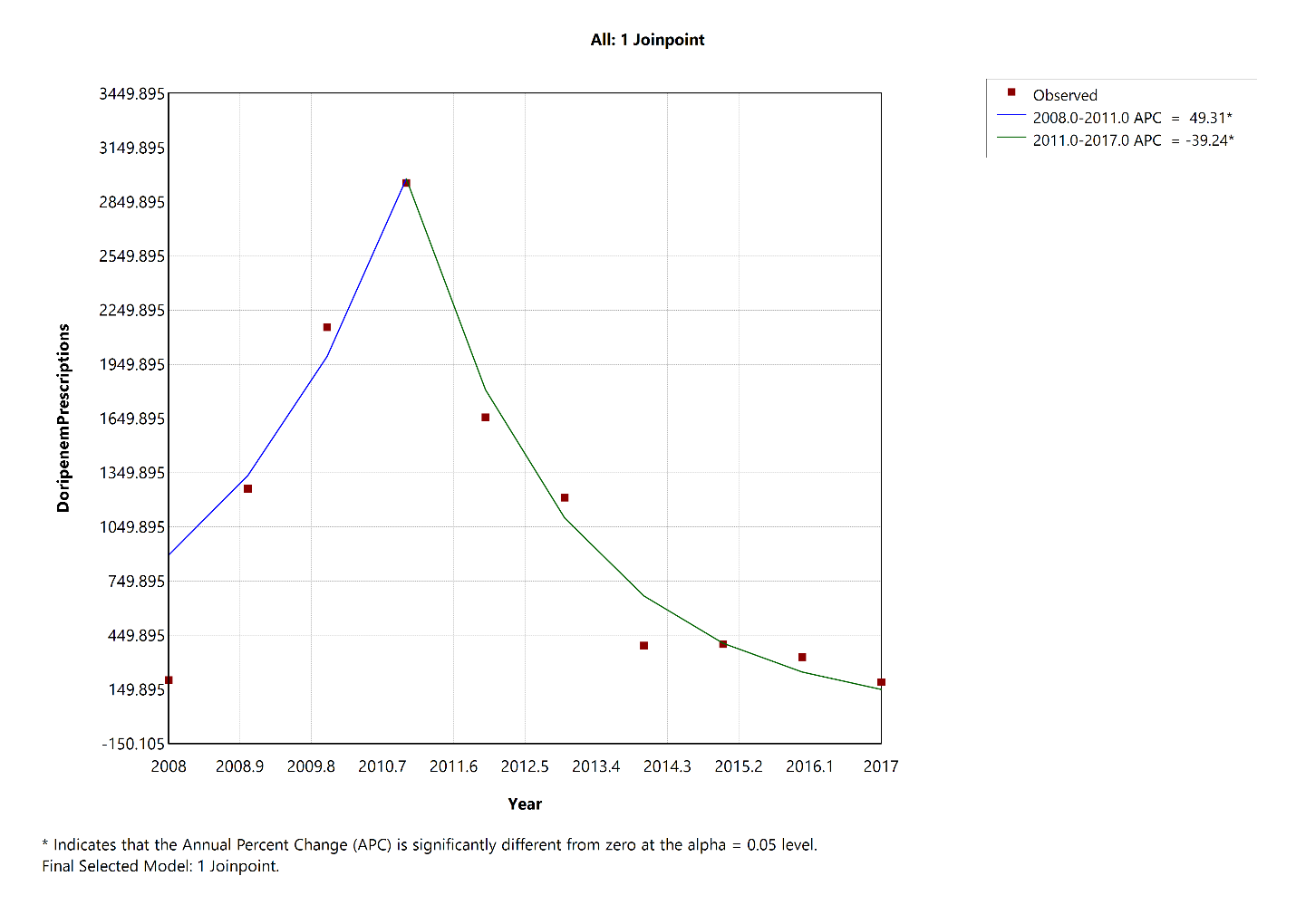

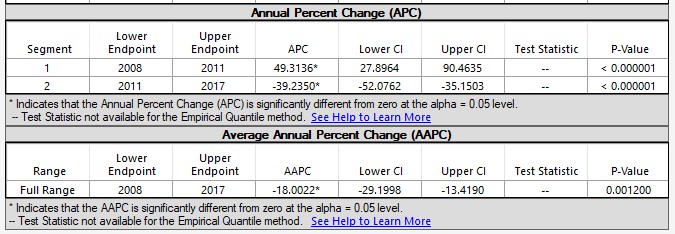


Figure S13: Join point regression of Doripenem reimbursement in Medicaid


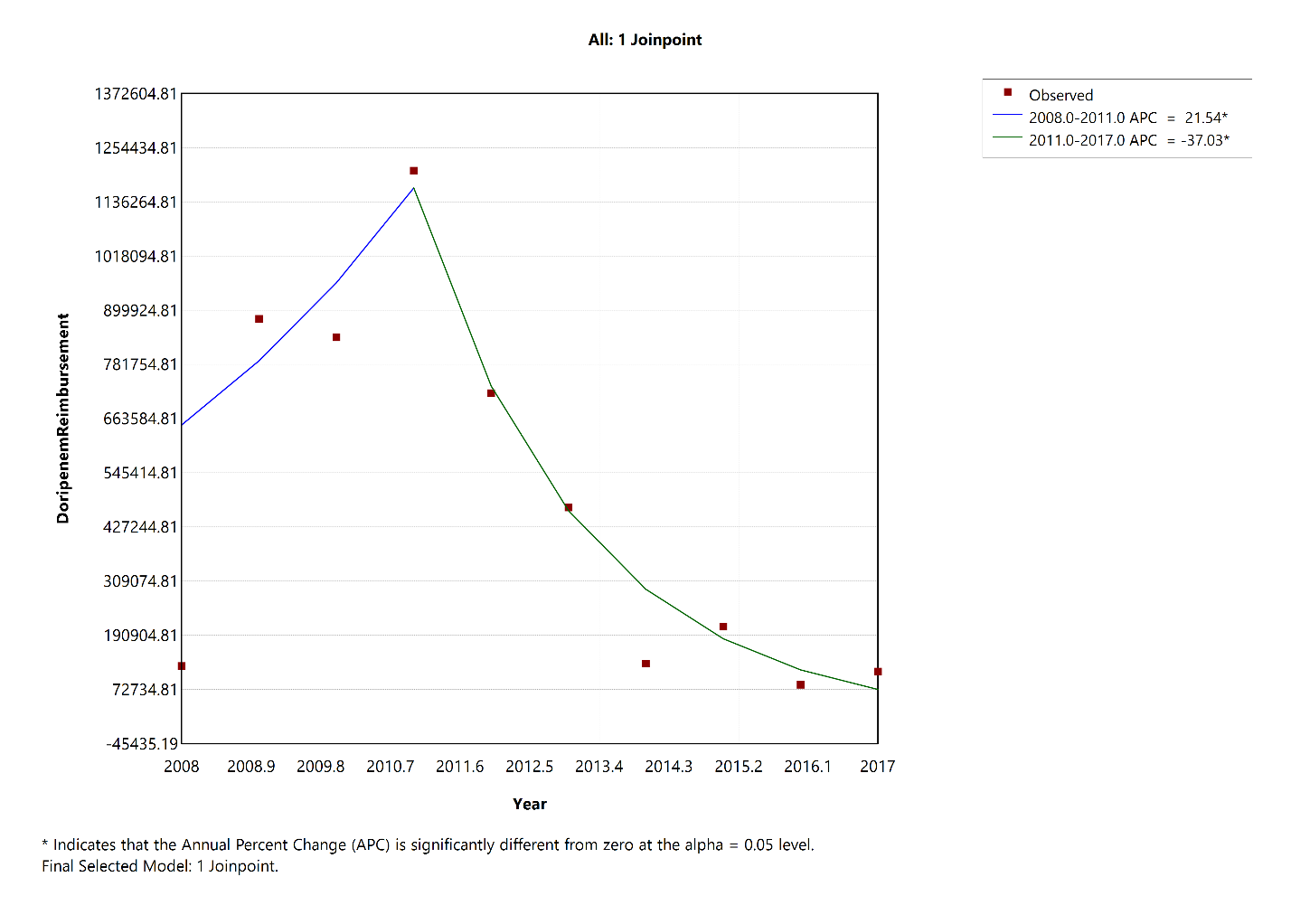

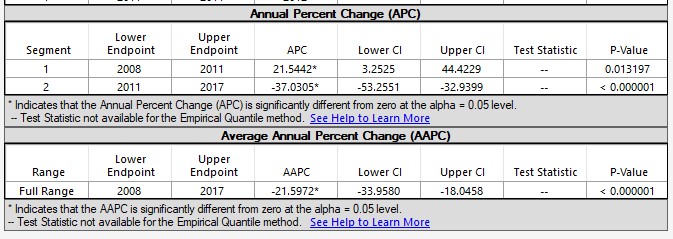


Figure S14: Join point regression of Doripenem proxy price in Medicaid


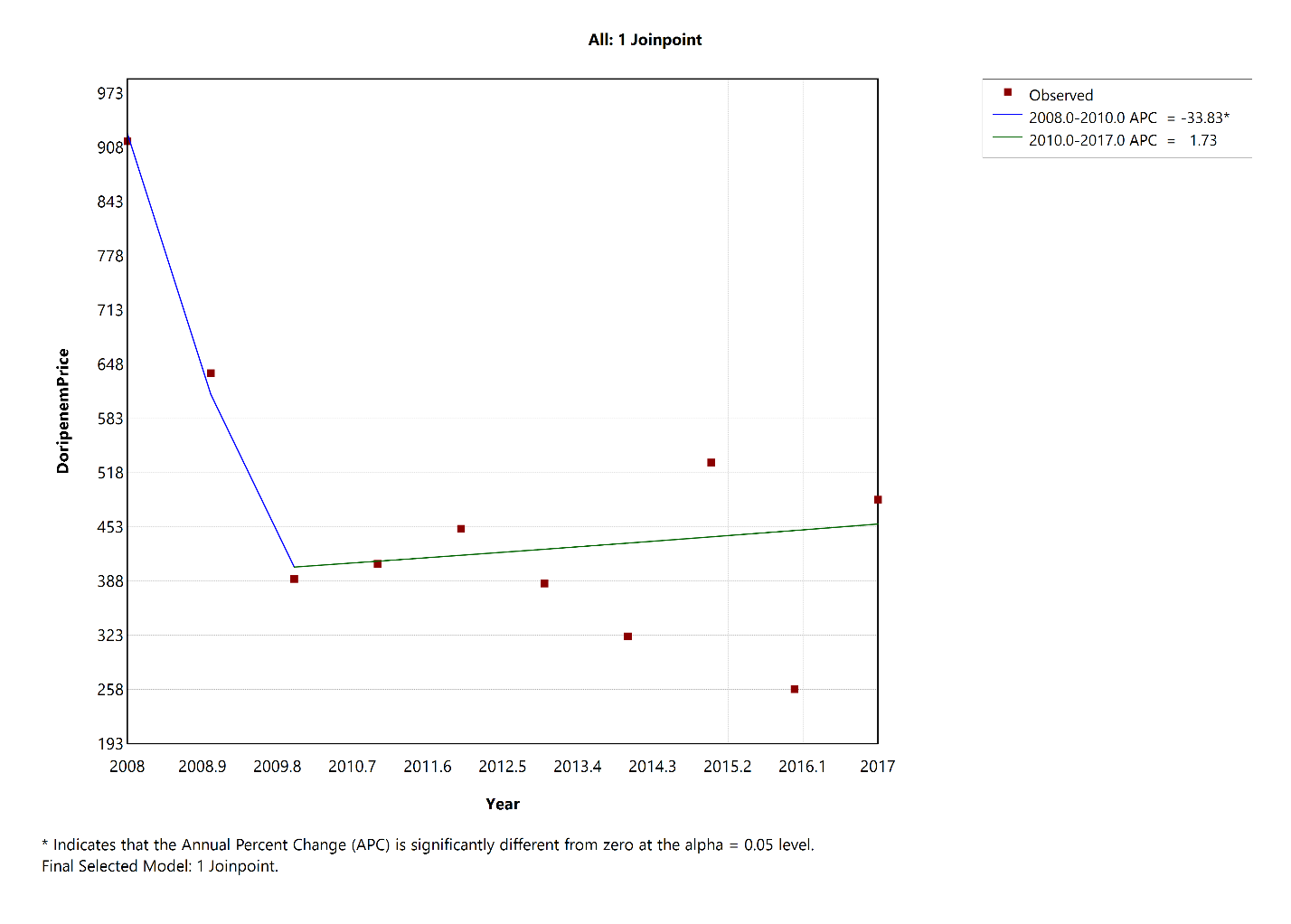

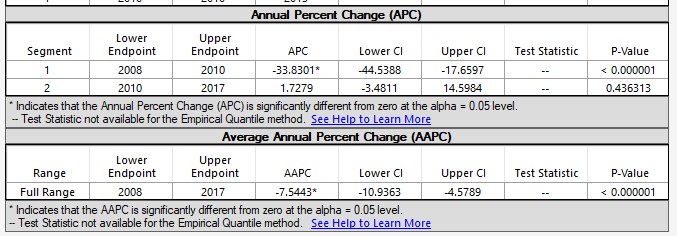

Supplement: Supplementary file 1 [file Data_Sheet_1.docx]
