# Supplementary material for: Trends in carbapenem antibiotics utilization, costs, and market dynamics in Medicaid: a retrospective analysis from 1991 to 2023
Source: Front Med (Lausanne). 2025 Aug 7;12:1589981. doi: 10.3389/fmed.2025.1589981 (PMC12367653; doi:10.3389/fmed.2025.1589981)
Supplement: Supplementary file 2 [file Table_1.docx]

Table 1: Trends in Carbapenem Utilization, Reimbursement, Pricing, and Market Share in Medicaid (1991–2023)
